# Construction and characterization of a functional chimeric laccase from metagenomes suitable as a biocatalyst

**DOI:** 10.1186/s13568-021-01248-y

**Published:** 2021-06-19

**Authors:** Nobuya Itoh, Yuya Hayashi, Serina Honda, Yuna Yamamoto, Daichi Tanaka, Hiroshi Toda

**Affiliations:** Biotechnology and Pharmaceutical Engineering Research Center and Department of Biotechnology, Toyama Prefectural University, 5180 Kurokawa, Imizu, Toyama 939-0398 Japan

**Keywords:** Metagenome, S-GAM technique, Multi-copper oxidase (MCO), Laccase, Chimeric enzyme, Biocatalyst

## Abstract

Screening of gene-specific amplicons from metagenomes (S-GAM) is an efficient technique for the isolation of homologous genes from metagenomes. Using the S-GAM approach, we targeted multi-copper oxidase (MCO) genes including laccase and bilirubin oxidase (BOX) in soil and compost metagenomes, and successfully isolated novel MCO core regions. These core enzyme genes shared approximately 70% identity with that of the putative MCO from *Micromonospora* sp. MP36. According to the principle of S-GAM, the N- and C-terminal regions of the deduced products of the mature gene come from the known parent gene, which should be homologous and compatible with the target gene. We constructed two different MCO hybrid genes using *Bacillus subtilis* BOX and *Micromonospora* sp. MP36 MCO, to give *Bs-mg-mco* and *Mic-mg-mco*, respectively. The constructed chimeric MCO genes were fused with the maltose-binding protein (MBP) gene at the N-terminus for expression in *Escherichia coli* cells. We found that MBP-Mic-mg-MCO/Mic-mg-MCO possessed the characteristic properties of laccase, although MBP-Bs-mg-MCO had no activity. This novel laccase (Mic-mg-MCO) demonstrated unique substrate specificity, sufficient activity at neutral pH, and high thermal stability, which are suitable properties for its use as a laccase biocatalyst.

**Supplementary Information:**

The online version contains supplementary material available at 10.1186/s13568-021-01248-y.

## Key points

We presented a series of modifications for the S-GAM approach, and successfully constructed functional chimeric laccase (Lcc) genes containing core regions from metagenomes.

## Introduction

Multi-copper oxidases (MCOs) are a family of enzymes that contain copper as a prosthetic group to catalyze four one-electron oxidations of various compounds concomitantly with the reduction of molecular oxygen to water (Sakurai and Kataoka 2007). MCOs are found in a wide range of organisms including bacteria, fungi (mainly laccase), plants, insects, and vertebrates (Baldrian 2006; Aniszewski et al. 2008; Dwivedi et al. 2011; Chauhan et al. 2017). They can oxidize various aromatic and non-aromatic compounds by a radical-catalyzed reaction mechanism that can undergo further oxidation or radical coupling reactions (Riva 2006). They generally contain three types of copper, of which type 1 is responsible for the blue color, oxidation of substrates, and electron extraction. The extracted electrons are transferred to type 2 and/or type 3 copper sites, where molecular oxygen is reduced to water (Sakurai and Kataoka 2007).

A broad range of substrates undergo laccase (Lcc) (EC 1.10.3.2) oxidation, including various phenolic and nonphenolic compounds, and the substrates overlap with those of monophenol monooxygenase tyrosinase (EC 1.14.18.1), catechol oxidase (1, 2-diphenol: dioxygen oxidoreductase, EC 1.10.3.1) (Aniszewski et al. 2008), and bilirubin oxidase (BOX) (EC 1.3.3.5) (Murao and Tanaka 1981; Tanaka and Murao 1982; Cracknell et al. 2011; Mano 2012). The range of substrates accepted as a hydrogen donor by Lcc is outstanding, and the oxidation of syringaldazine in combination with an inability to oxidize tyrosine and bilirubin is a recognized indicator of Lcc activity (Baldrian 2006; Mano 2012). Accordingly, due to the ability of Lccs to catalyze electron transfer reactions without additional cofactors, they play important roles in the textile industry (Shraddha et al. 2011), pulp industry (Sigoillot et al. 2002), food industry (Brijwant 2010; Itoh et al. 2017), biodegradation of environmental pollutants (Camarero et al. 2008), and synthetic chemistry (Riva 2006; Mogharabi and Faramarzi 2014; Díaz-Rodríguez et al. 2014).

The screening of gene-specific amplicons from metagenomes (S-GAM) approach we have developed is a powerful technique for the efficient isolation of target genes from metagenomes (Itoh et al. 2014, 2016b; Itoh 2017). This approach can overcome a major disadvantage of previous techniques, namely, low efficiency in obtaining target genes from metagenomes. S-GAM also permits the omission of time-consuming subcloning and expression-optimization procedures. Simply put, S-GAM is a unique shortcut to identify proteins hidden in nature. However, this method yields gene products that are all chimeric at the N- and C-terminal regions; thus, resulting in improper folding of the protein in some conditions and the need for further improvements of the enzymes by changing or optimizing these regions.

In this study, we describe the isolation of core DNA regions of novel MCO genes from soil metagenomes, optimization of the N- and C-terminal regions, expression of this chimeric Lcc gene in *Escherichia coli*, and characterization of this novel Lcc. Moreover, we present a series of modifications for the S-GAM approach to obtain a functional enzyme.

## Materials and methods

### Preparation of metagenomes from soil samples

Metagenomic DNA was extracted from 14 environmental samples collected from soils, farm (35–45 °C) and bark (50–80 °C) composts, and activated sludge and its compost in Toyama and Kagoshima, Japan, using an ISOIL for Beads Beating™ Kit (Nippon Gene, Tokyo, Japan) without further purification. Bark compost samples fermented at 50–80 °C were generously supplied by a compost-producing company (Hokuriku Port Service, Toyama, Japan). Successful extraction of DNA from soil and compost samples was confirmed using agarose gel electrophoresis, and DNA concentration was measured by NanoVue (GE Healthcare Life Sciences, Buckinghamshire, UK); these DNA samples served as templates for PCR.

### Genome database homology search and design of PCR primers

**Fig. 1:**
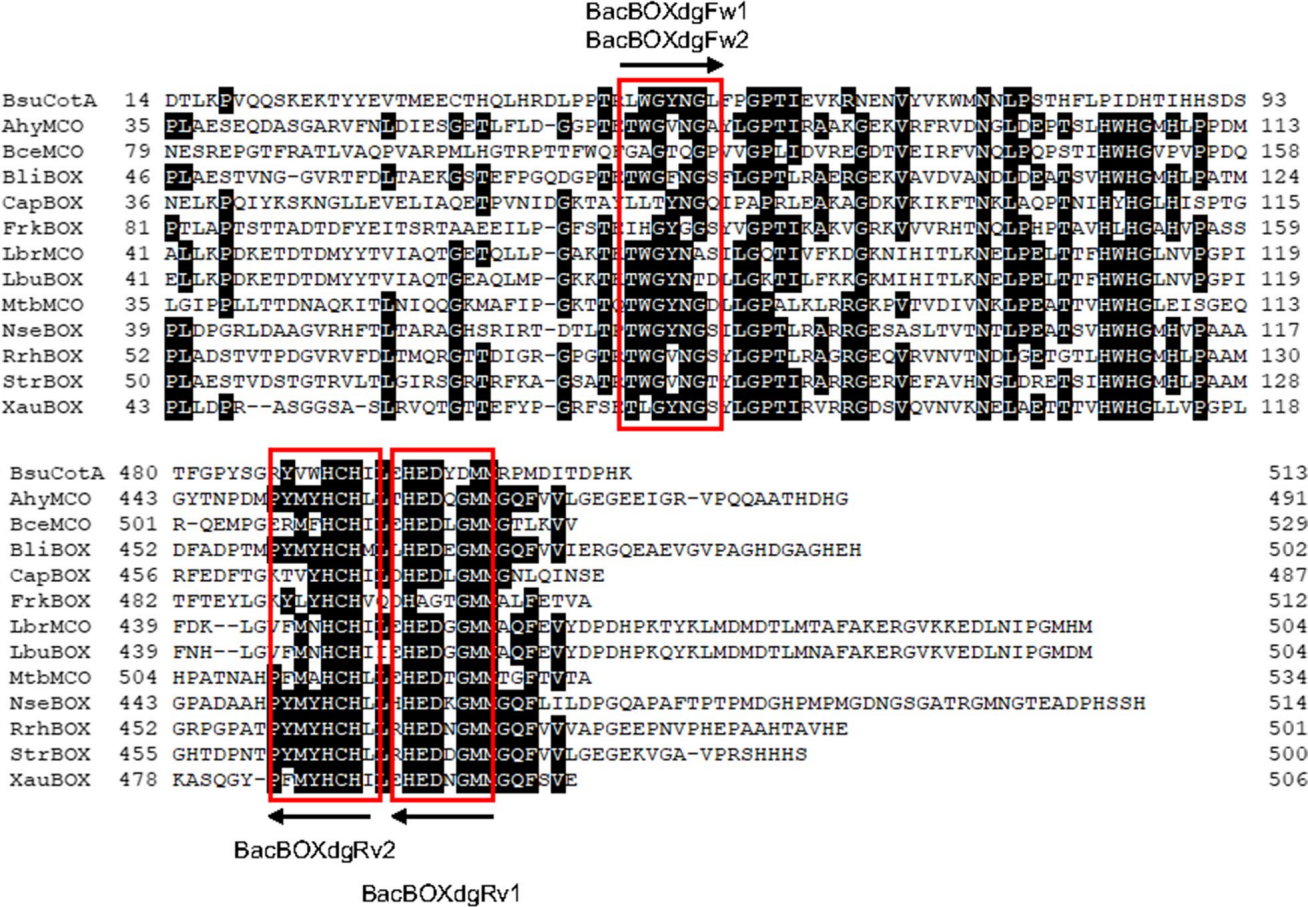
Alignment analysis of several MCOs to design the primers for isolating target genes from metagenomes. Amino acid sequences were aligned using ClustalW and conserved amino acids were denoted by white letters on black boxes. The conserved amino acid sequences surrounded by a red square were used for design of each primer. *B. subtilis* (BsuCosA; GenBank accession No.: CAB12449.1), *Actinoalloteichus hymeniacidonis* (AhyMCO; AOS64008), *Burkholderia cepacia* (BceMCO; AFQ47357), *Brevibacterium linens* (BliBOX; KHS53242), *Cyanobacterium aponinum* (CapBOX; AFZ53266), *Frankia* sp. (FrkBOX; ABW14182), *Lactobacillus brevis* (LbrMCO; EEI71081), *L. buchneri* (LbuBOX; AEB74417), *Mycobacterium tuberculosis* (MtbMCO; SGC69794), *Nocardia seriolae* (NseBOX; APA98799), *Rhodococcus rhodochrous* (RrhBOX; ETT23846), *Streptomyces* sp. (StrBOX; AEN08529), *Xanthobacter autotrophicus* (XauBOX; ABS68135)

A database homology search was performed using BLAST (National Center for Biotechnology Information, Bethesda, MD, USA). Bacterial BOX genes whose products shared 40–80% amino acid sequence identity with *Bacillus subtilis* CotA (Lcc/BOX, accession no. CAB12449.1) (Hullo et al. 2001) were selected from databases, and the homologous regions as near as possible to the N- and C-terminal regions were determined (Fig. 1). Subsequently, two sets of forward and reverse degenerate primers were designed: BacBOXdgFw1, BacBOXdgFw2, BacBOXdgRv1, and BacBOXdgRv2 (Table 1). The following GAM primers for PCR were designed to introduce 15-bp fusion sites to both 5′-ends: BacBOXdgFw1GAM, BacBOXdgFw2GAM, mgBOXInfFw, and mgBOXInfRv (Table 1).

**Table 1:** PCR primers for isolating the core genes of MCO from metagenomes

Primer	Sequence (5′-3′)
BacBOXdgFw1	ACNTGGGGNTAYAAYGGNWS
BacBOXdgFw2	ACNTGGGGNGTNAAYGGNWS
BacBOXdgRv1	CATCATNCCNYYRTCYTCRTG
BacBOXdgRv2	ADRTGRCARTGRTACATRWA
BacBOXdgFw1GAM	CTGCCGCCGACCCGT ACNTGGGGNTAYAAYGGNWS
BacBOXdgFw2GAM	CTGCCGCCGACCCGT ACNTGGGGNGTNAAYGGNWS
BacBOXdgRv1GAM	GATATCCATCGGACG CATCATNCCNYYRTCYTCRTG
BacBOXdgRv2GAM	GACCTCATGCTCCAG ADRTGRCARTGRTACATRWA
BacBOXInfFw	CGTCCGATGGATATCACCG
BacBOXInfRv	ACGGGTCGGCGGC
MicMCOInfFw	GCTGCGCCATGAGGAT
MicMCOInfRv	CAGCGTTTTGCGATCGC
MicMCOFwbam	AGACGGATCCAGCATTCAACCAGTGGCC
mgBOXInfFw	GATCGCAAAACGCTGACNTGGGGNTAYAAYGGNWS
mgBOXInfRv	TCCTCATGGCGCAGCADRTGRCARTGRTACATRWA
MicMCOFw	ATGAGCATTCAACCAGTGGC
pMALdelMBPRv	GGCGAGAGCCGAGGC

### PCR amplification of target genes

Standard techniques were used for DNA manipulation (Sambrook and Russell 2001). *E. coli* JM109, *E. coli* BL21 (DE3), and *E. coli* 10-β (New England Biolabs, Tokyo, Japan) were used to host *mco* and *lcc* genes***.*** PCR was performed using KOD FX Neo DNA polymerase (Toyobo, Osaka, Japan). Hot-start and step-down PCR protocols were used to avoid non-specific amplification. Each reaction mixture contained 10 µL of 2× buffer from the KOD FX Neo kit, 2 nmol of each dNTP, 8 pmol of each primer, 10–50 ng metagenomic DNA, and 0.4 U DNA polymerase in a total volume of 20 µL. PCR commenced at 94 °C for 2 min, followed by a step-down protocol: 4 cycles at 94 °C for 10 s, 56 °C for 30 s, and 68 °C for 1 min; 4 cycles at 94 °C for 10 s, 54 °C for 30 s, and 68 °C for 1 min; 4 cycles at 94 °C for 10 s, 52 °C for 30 s, and 68 °C for 1 min; 4 cycles at 94 °C for 10 s, 50 °C for 30 s, and 68 °C for 1 min; 20 cycles at 94 °C for 10 s, 48 °C for 30 s, and 68 °C for 1 min; and finally the sample was maintained at 68 °C for 5 min. The obtained amplicons were analyzed by agarose gel electrophoresis. Following confirmation of the proper amplification of the target genes, the GAM-primer sets were applied to these positive metagenomic samples. The same PCR conditions were used for the GAM-primer sets, except that the following step-down annealing temperatures were applied: 58 °C, 56 °C, 54 °C, 52 °C, and 50 °C. The gene-specific amplicons obtained with the GAM-primer sets were purified using a FastGene Gel/PCR Extraction Kit (Nippon Genetics, Tokyo, Japan) from the agarose gel after electrophoresis.

### Construction of expression vectors

*B. subtilis* CotA (Lcc/BOX) and *Micromonospora* sp. MP36 MCO (accession no. WP_148798198.1) genes were chemically synthesized to optimize translational codon usage in *E. coli* as the pUC57-Kan-BsBOX and pUC57-Kan-MicMCO plasmids, respectively, by GENEWIZ® Japan (Saitama, Japan). Each plasmid, isolated from recombinant *E. coli* JM109 cultured in Luria–Bertani (LB) medium (1% [w/v] tryptone, 0.5% [w/v] yeast extract, and 0.5% [w/v] NaCl; pH 7.0) with 50 µg/mL kanamycin at 37 °C overnight, was purified using a FastGene Plasmid Mini Kit (Nippon Genetics), digested with *Nde*I and *Bam*HI, and further purified from an agarose gel using a FastGene Gel/PCR Extraction Kit. The purified gene was ligated between the same restriction enzyme sites in the multiple cloning site of the pMAL-c5x plasmid (New England Biolabs, Tokyo, Japan) to give pMAL-c5x-BsBOX and pMAL-c5x-MicMCO, respectively. pMAL-c5x-BsBOX can normally express the *box* gene as a maltose-binding protein (MBP)-fusion protein in *E. coli* BL21 (DE3), and it can be used as a BOX/Lcc-positive clone.

Approximately 900 bp was deleted from the inner part of *Bsbox* in pUC57-Kan-BsBOX and *Micmco* in pUC57-Kan-MicMCO by inverse PCR using a KOD-Plus-Mutagenesis Kit (Toyobo). After amplification by inverse PCR, the linearized plasmids were self-ligated, and they were transformed into *E. coli* JM109 by electroporation. Following cultivation in LB medium (pH 7.0) with 50 µg/mL kanamycin at 37 °C overnight, the plasmids were extracted from the recombinant cells, the deleted DNA fragment of *Bsbox* or *Micmco* treated with *Nde*I and *Bam*HI was separated by agarose gel electrophoresis and purified from the gel using a FastGene Gel/PCR Extraction Kit. The purified DNA fragments (ca. 400 bp) were ligated between the same restriction enzyme sites of pMAL-c5x to generate pMAL-c5x-delBsBOX and pMAL-c5x-delMicMCO, respectively.

Using pMAL-c5x-delBsBOX or pMAL-c5x-delMicMCO as a template, inverse PCR was carried out to linearize and insert 15 nucleotides at both 5′-ends for the In-Fusion reaction using the following primer sets: BacBOXInfFw and BacBOXInfRv for pMAL-c5x-delBsBOX; and MicMCOInfFw and MicMCOInfRv for pMAL-c5x-delMicMCO. After PCR amplification, the linearized plasmids were purified using a FastGene Gel/PCR Extraction Kit and used for the In-Fusion reaction.

The gene-specific amplicons containing the core BOX/MCO gene were fused between the same compatible sites of linearized pMAL-c5x-delBsBOX or pMAL-c5x-delMicMCO using an In-Fusion® HD Cloning Kit (Clontech, Mountain View, CA, USA) to generate each expression vector. The reaction mixture consisted of 2 µL In-Fusion HD enzyme premix, 2 µL DNA fragment amplified from pMAL-c5x-delBsBOX or pMAL-c5x-delMicMCO (ca. 50 ng in total), 2 µL DNA from metagenomes (ca. 50 ng in total), 2 µL In-Fusion enzyme, and 2 µL sterilized water in a total volume of 10 µL, and the mixture was incubated at 50 °C for 15 min. In accordance with the manufacturer’s protocol, the obtained plasmids (2 µL) were transformed into a suspension of *E. coli* 10-β (50 µL) by heat-shock (pre-incubation at 0 °C for 30 min, 42 °C for 30 s, and incubation at 0 °C for 5 min), and recovered in New England Biolabs 10-β stable outgrowth medium at 37 °C for 1 h. The clones were grown overnight at 37 °C on agar plates containing LB medium (pH 7.0) with 50 µg/mL ampicillin. Clones grown on the agar plates were selected randomly and the plasmids were extracted. The plasmids, designated as pMAL-c5x-Bs-mg-MCO and pMAL-c5x-Mic-mg-MCO, were transformed into *E. coli* BL21 (DE3)/pG-KJE8 (chaperone plasmid; Takara Bio Inc., Shiga, Japan) by electroporation.

To construct the MBP-deleted Mic-mg-MCO (Mic-mg-MCOΔMBP), inverse PCR was performed using pMAL-c5x-Mic-mg-MCO120-3 as a template with the MicMCOFw2 and pMALdelMBPRv primer set (Table 1). The amplified fragment was treated with *Dpn*I and self-ligated to give pMAL-c5x-Mic-mg-MCO120-3ΔMBP. The deletion of MBP was confirmed by plasmid analysis on agarose gel electrophoresis and DNA sequencing.

DNA sequences were determined for both strands using a capillary DNA sequencer (ABI PRISM 310; Applied Biosystems® Life Technologies, Carlsbad, CA, USA).

### Screening for Lcc activity in *E. coli*

Screening for Lcc activity in recombinant *E. coli* BL21 (DE3) harboring the pMAL-c5x-Bs-mg-MCO or pMAL-c5x-Mic-mg-MCO/pG-KJE8 chaperone plasmid was performed on agar plates consisting of LB medium (pH 7.0) with 1 mM of 2,6-dimethoxyphenol (DMP), 0.5 mM CuSO_4_, 0.1 mM isopropyl-β-thiogalactopyranoside (IPTG), 50 µg/mL ampicillin, 20 µg/mL chloramphenicol, 5 ng/mL tetracycline, 0.5 mg/mL arabinose, and 1.5% agar, in which tetracycline and arabinose are necessary to induce chaperones from pG-KJE8. Brown from DMP oxidation developed around the positive colonies. Spectrophotometric measurements of 2,2′-azino-bis-(3-ethylthiazoline-6-sulphonate) (ABTS) activity were also performed for recombinant cells, which had been cultured at 37 °C overnight with shaking in LB liquid medium (0.5 mL) containing 50 µg/mL ampicillin, 20 µg/mL chloramphenicol, 5 ng/mL tetracycline, and 0.5 mg/mL arabinose in 96-well deep plates, and then 0.1 mM IPTG and 1 mM CuSO_4_ were added to the culture medium to induce enzyme expression, and cultured for a further 18 h at 18 °C. After collecting and washing the cells with 50 mM potassium phosphate buffer (KPB, pH 7.0), ABTS (10 mM) was added to the cell suspension (0.3 mL) in the buffer, incubated at 25 °C overnight with shaking at 1,000 rpm (BioShaker MBR-022UP, Taitec, Saitama, Japan), and development of a clear blue was observed for positive clones.

### Enzyme assay

The reaction mixture (1 mL) consisted of 50 mM KPB (pH 7.0) containing 10 mM DMP or 10 mM of all other substrates, except for syringaldazine (5 mM). Generally, Lcc activity was assayed spectrophotometrically at 30 °C by measuring the increase in the absorbance of 2,2′,6,6′-tetramethoxydibenzo-1,1′-diquinone (dimeric DMP) at 468 nm (ε = 49.6 mM^−1^ cm^−1^) from DMP or oxidation of ABTS at 420 nm (ε = 36.0 mM^−1^ cm^−1^), the result of which was used to calculate activity. One unit of enzyme was defined as the amount that converted 1 μmol of substrate in 1 min under these conditions. Similarly, the following absorption coefficients for the oxidized compound of the substrate were used to calculate activity [nm, ε (mM^−1^ cm^−1^)]: syringaldazine (525, 65), *o*-guaiacol (465, 12), *o*-catechol (412, 2.2), and pyrogallol (420, 4.4). BOX activity was measured at 30 °C as a decrease in absorbance at 440 nm of bilirubin (20 μg/mL; ε = 25 mM^−1^ cm^−1^) in 50 mM Tris–HCl buffer (pH 7.5) due to the low solubility of bilirubin at low pH (Durand et al. 2012). Activity toward l-tyrosine (475 nm), l-3,4-dihydroxyphenylalanine (l-DOPA) (475 nm), and gallic acid (420 nm) was regarded as an increase in absorbance at each wavelength for the oxidized compound [polyphenol oxidase unit (POU)]; 1 POU indicates a 1.0 increase in absorbance in 1 min at 30 °C. Iodide oxidation activity was measured according to the method of Amachi et al. (2005) as the formation of molecular I_2_ from iodide anions.

### Purification and physicochemical characterization of chimeric Lcc

Mic-mg-MCO120-3 was purified at 0–4 °C in 20 mM KPB (pH 7.0) unless otherwise stated. Washed recombinant *E. coli* BL21 (DE3)/pMAL-c5x-Mic-mg-MCO120-3ΔMBP/pG-KJE8 cells from 500 mL culture, which were cultured at 37 °C for 17 h in LB medium in a 2-L shake flask and then at 18 °C for a further 16 h with 0.1 mM IPTG and 1 mM CuSO_4_, were washed with the buffer, suspended in 30 mL buffer, and disrupted using an ultrasonic oscillator (Ultra Sonic Disrupter UD-200; Tomy Corp., Tokyo, Japan) for 150 s (5 disruption sequences of 30 s followed by a 60-s interval for cooling). After centrifugation (12,000×*g*, 15 min), the cell-free extract was fractionated with solid ammonium sulfate. The precipitate with 0 to 40% saturation of ammonium sulfate was collected, dialyzed against the buffer, and then applied to a DEAE-Toyopearl 650 M (Tosoh Co., Ltd., Tokyo, Japan) column (20 × 100 mm) equilibrated with the buffer. The column was washed with 10 column volumes of the buffer. Mic-mg-MCO120-3 was eluted with a linear 0–0.5 M NaCl gradient in the same buffer. Fractions exhibiting high levels of enzyme activity at approximately 0.2 M NaCl were collected and dialyzed against the buffer. The enzyme solution obtained was used as purified enzyme.

Protein concentration was estimated by measuring the absorbance of protein-containing solutions at 280 nm or by the method of Bradford using bovine serum albumin as a standard (Protein Assay Kit; Bio-Rad, Hercules, CA, USA). Sodium dodecyl sulfate-polyacrylamide gel electrophoresis (SDS-PAGE) was performed using 12% (w/v) polyacrylamide slab gels and the Tris–glycine buffer system of Laemmli (1970). The molecular weight of the purified enzymes was determined by analytical high-performance liquid chromatography using a TSK-Gel G3000SW_XL_ (Tosoh, Tokyo, Japan) column (7.8 mm × 30 cm) and an elution flow rate of 0.5 mL/min in 50 mM Tris–HCl (pH 7.0) with 0.1 M NaCl. The molecular mass of the enzyme was determined by comparing its retention time with those of standard proteins.

### Chemicals

pMAL-c5x was purchased from New England Biolabs (Tokyo, Japan). DNA-manipulating reagents were supplied by TOYOBO (Osaka, Japan) and Takara Bio (Shiga, Japan). ABTS was purchased from Sigma-Aldrich (Tokyo, Japan), and other chemicals were obtained from FUJIFILM Wako Chemicals (Osaka, Japan) or Tokyo Kasei (Tokyo, Japan).

### Nucleotide sequence accession number

The *lcc120-3* (*Mic-mg-mco120-3Δmbp*) sequence was registered at the DNA Data Bank of Japan under the accession number LC624142.

## Results

### Design of primers and amplification of *mco* genes by S-GAM

Degenerate primers were designed according to the N- and C-terminal regions of bacterial BOX and its related genes, belonging to the MCO family, which shared 40–80% identity with *B. subtilis* CotA (BOX/Lcc), and are proteins of approximately 513 amino acid residues (Fig. 1). However, alignment of several MCOs at the terminal regions showed quite low identity to each other, and the well-conserved regions were located in the more inner regions from the N- and C-termini (Fig. 1), which were TWG(Y/V)NGS in the N-terminal region and (F/Y)MYHCH(I/L) and HED(G/N)GMM in the C-terminal region. Primers of approximately 20 bp (BacBOXdgFw1/Fw2, BacBOXRv1, and BacBOXRv2) were designed based on these sequences. GAM-primers of approximately 35 bp (BacBOXdgFw1GAM, BacBOXdgFw2GAM, BacBOXdgRv1GAM, and BacBOXdgRv2GAM), containing the regions for fusion with the pMAL-c5x-delBsBOX expression vector, were also designed (Table 1). First, a combination of the primers BacBOXdgFw1/Fw2 and BacBOXRv1/BacBOXRv2 was tested to amplify the core genes for some metagenomes. Adjustments were made to the primer set combinations and to optimize other PCR conditions, especially annealing temperatures. We used hot-start and step-down PCR protocols to avoid nonspecific amplification and to support the sufficient amplification of DNA using KOD FX Neo DNA polymerase. Under the optimized conditions, we observed the amplification of target genes from 8 samples among 14 metagenomic samples, which were mainly isolated from bark composts under fermentation collected in Japan. Second, we carried out amplification using the optimized GAM-primer set (BacBOXdgFw1GAM and BacBOXdgRv2GAM) based on the first amplification results to isolate the core DNAs of the target genes and to fuse them to the expression vector. This simple method was effective to determine which metagenomic samples should be used to amplify the target genes with the GAM-primer set.

### Screening and analyses of *mco* genes from the metagenomic library

PCR-amplified genes with the GAM-primer set were fused with the parent gene, *Babox*, at both terminal regions using a linearized DNA fragment amplified from pMAL-c5x-delBsBOX. They were transferred into *E. coli* and expressed as MBP-fusion proteins because it is difficult to express many *mco* genes normally in *E. coli* cells. In advance of this experiment, we had confirmed that MBP-BsBOX can be expressed as an active form in *E. coli* BL21 (DE3) with the pMAL-c5x-BsBOX/pG-KJE8 system. Approximately 2000 colonies obtained from 4 compost metagenomes were measured directly using their enzyme activity for two substrates, DMP on agar plates and ABTS in liquid medium. However, all clones were null for enzymatic activity, despite observing normal protein expression in some clones by SDS-PAGE (data not shown). Therefore, we analyzed the 12 DNA sequences of the potential *mco* genes obtained from 4 compost metagenomes, and the results indicated that most core DNAs had high similarity with the putative MCOs of *Micromonospora* sp. MP36 (ca. 70% amino acid identity) and *Comamonadaceae* (accession no. TY22925.1) (ca. 60% amino acid identity), but relatively low similarity with that of BsBOX (ca. 30% amino acid identity) (Table 2). The chimeric enzymes, except MBP, consisted of 49 amino acid residues of BsBOX at the N-terminus, 387 residues of the core region, and 22 residues of BsBOX at the C-terminus in a total of 458 amino acid residues, suggesting that the N- and C-amino acid residues are incompatible with the core region of the enzymes.

**Table 2:** BLAST analysis of MCO core gene DNAs isolated from metagenomes

Clone no	Description of known enzyme	Accession no	Amino acid identity to known MCO (%)
s120-2	MCO domain-containing protein (*Micromonospora* sp. MP36)	WP_148798198.1	73.2
s120-3	MCO domain-containing protein (*Micromonospora* sp. MP36)	WP_148798198.1	72.8
s120-4	MCO domain-containing protein (*Micromonospora* sp. MP36)	WP_148798198.1	72.9
s120-5	MCO domain-containing protein (*Micromonospora* sp. MP36)	WP_148798198.1	71.9
s121-1	MCO domain-containing protein (*Micromonospora* sp. MP36)	WP_148798198.1	72.7
s123-1	MCO domain-containing protein (*Micromonospora* sp. MP36)	WP_148798198.1	72.6
s123-3	MCO (*Comamonadaceae*)	RYF17308.1	60.2
s123-4	MCO domain-containing protein (*Micromonospora* sp. MP36)	WP_148798198.1	72.9

To obtain an active form of MCO, we substituted the N- and C-terminal regions of BsBOX with those of MicMCO. Eight clones shown in Table 2 isolated from three bark compost samples (s-120, s-121, and s-123) and eliminated the sequence-duplicated ones, which showed high similarity with MicMCO, were PCR-amplified using the mgBOXInfFw and mgBOXInfRv primer set (Table 1), and fused to the linearized DNA fragment amplified from pMAL-c5x-delMicMCO. Amino acid sequence identity, as compared to that of MicMCO, for the core regions by BLASTP analysis is summarized in Table 2. The hybrid genes were given the descriptor *mbp-Mic-mg-mco* to indicate the chimeric MCO of *Micromonospora* sp. and metagenomic MCO. The chimeric enzymes, except MBP, consisted of 98 amino acid residues of MicMCO at the N-terminus, 387 residues of the core region, and 54 residues of MicMCO at the C-terminus in a total of 539 amino acid residues. The core regions had 98–99% amino acid identity to each other except 123-3 (Additional file 1). The activity of these clones after inducing gene expression was measured spectrophotometrically using cell-free extracts from recombinant *E. coli*. We confirmed they had measurable Lcc activity, although there was a large difference among their activity (Table 3). We observed that the MBP-Mic-Mg-MCO120-3 clone possessed fourfold higher activity than MBP-MicMCO. Moreover, some amino acid substitutions (Additional file 1) in the core region significantly affected the enzyme activity of MBP-Mic-mg-MCOs.

**Table 3:** Activity of the constructed Lccs (MBP-Mic-mg-MCO) for ABTS and DMP

Clone	Specific activity for ABTS and DMP (mU/mg protein)^a^	
	ABTS	DMP
MBP-Mic-MCO	11.0	N.D.^2^
MBP-Mic-mg-MCO120-2	13.54	2.48
MBP-Mic-mg-MCO120-3	40.88	8.05
MBP-Mic-mg-MCO120-4	3.08	0.07
MBP-Mic-mg-MCO120-5	0.05	N.D
MBP-Mic-mg-MCO121-1	4.24	0.07
MBP-Mic-mg-MCO123-1	9.12	0.32
MBP-Mic-mg-MCO123-3	0.01	N.D
MBP-Mic-mg-MCO123-4	3.30	0.26

^a^Activity in the crude extract of recombinant *E. coli*

*N.D.* not detected

For the application of MBP-Mic-mg-MCO120-3 as a biocatalyst, we tried to eliminate MBP from MBP-Mic-mg-MCO120-3 to generate Mic-mg-MCO120-3 by inverse PCR from pMAL-c5x-Mic-mg-MCO120-3, resulting in pMAL-c5x-Mic-mg-MCO120-3ΔMBP. We observed that *E. coli* BL21 (DE3) with pMAL-c5x-Mic-mg-MCO120-3ΔMBP/pG-KJE8 were able to stably produce Lcc120-3 (Mic-mg-MCO120-3), although enzyme productivity was unstable in the absence of the pG-KJE8 chaperone plasmid.

### Production and purification of chimeric Lcc from recombinant *E. coli*

Enzyme production by recombinant *E. coli* BL21 (DE3)/pMAL-c5x-Mic-mg-MCO120-3ΔMBP/pG-KJE8 cells was optimized. Enzyme production was induced with 0.1 mM IPTG and 1 mM CuSO_4_ at 18 °C for 16 h following cultivation in LB medium containing 50 µg/mL ampicillin, 20 µg/mL chloramphenicol, 5 ng/mL tetracycline, and 0.5 mg/mL arabinose at 37 °C for 17 h in a shake flask. Enzyme production reached 27–47 mU (DMP) and 90–160 mU (ABTS)/mL culture medium.

**Fig. 2:**
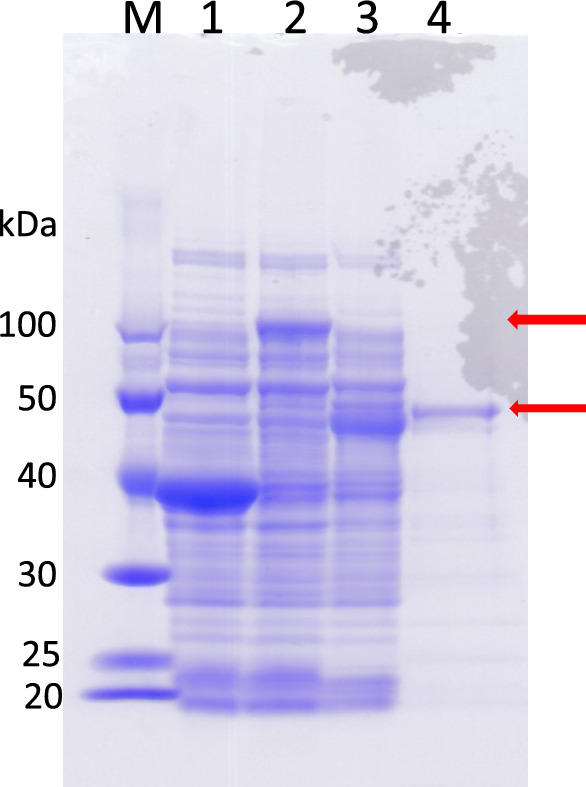
SDS-PAGE analysis of recombinant MBP-Lcc120-3 (MBP-Mic-mg-MCO) and Lcc120-3 without MBP (Mic-mg-MCO), which were expressed in *E. coli* BL21 (DE3). Lane M, molecular weight standards (Pre-stained XL-Ladder; APRO Science, Tokushima, Japan); lanes 1, 2, and 3, crude enzyme solutions from recombinant *E. coli* (pMAL-c5x/pG-KJE8, pMAL-c5x-Mic-mg-MCO120-3/pG-KJE8, and pMAL-c5x-Mic-mg-MCO120-3ΔMBP/pG-KJE8, respectively); lane 4, purified Lcc120-3

The enzyme was relatively stable during purification, and also towards concentrated NaCl. Therefore, the process of purifying Lcc120-3 (Mic-mg-MCO120-3) from the crude extract was conducted by ammonium sulfate fractionation and anion-exchange chromatography using DEAE-Toyopearl. However, total enzymatic activity was largely lost with anion-exchange chromatography including DEAE- or QAE-resin. The reason for this phenomenon is unclear. The enzyme was purified by 10.8-fold, and the yield was 13.2%. SDS-PAGE indicated the purified enzyme preparation was almost homogeneous (Fig. 2).

### Enzymatic properties of chimeric Lcc

#### Molecular mass and subunit structure

The molecular mass of Lcc120-3 (Mic-mg-MCO120-3) was estimated to be 57 kDa according to analytical high-performance liquid chromatography with a TSK-Gel G3000SW column. SDS-PAGE revealed an almost single band corresponding to a molecular mass of 50 kDa (Fig. 2), indicating that Lcc120-3 is a monomeric enzyme. As described, the enzyme consisted of 539 amino acid residues. Therefore, the theoretical molecular mass of Lcc120-3 was inferred to be 58.4 kDa. The *pI* value of Lcc120-3 estimated from the amino acid sequence was 5.92. On the other hand, the theoretical molecular mass of MBP-LCC120-3 was estimated to be 106.6 kDa, including MBP (48.2 kDa) and chimeric Mic-mg-MCO (58.4 kDa). The molecular mass observed on SDS-PAGE matched the theoretical one (Fig. 2).

#### Effect of pH on enzyme activity

**Fig. 3:**
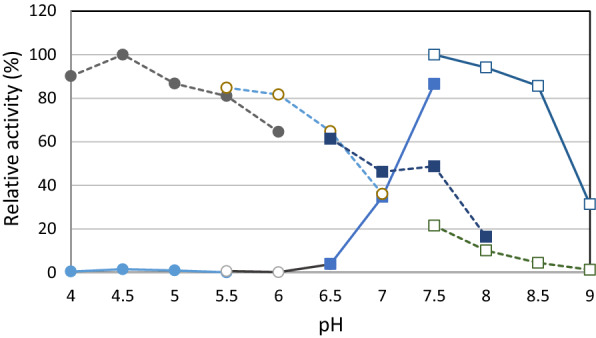
Effect of pH on the activity of purified Lcc120-3 (Mic-mg-MCO) for ABTS and DMP. Closed circles indicate acetate buffer (pH 4.0–6.0), open circles indicate citrate-NaOH buffer (pH 5.5–7.0), closed squares indicate KPB (pH 6.5–8.0), and open squares indicate Tris–HCl buffer (pH 7.5–9.0). The dotted line indicates the activity for ABTS, and the solid line indicates the activity for DMP. Relative activity is shown as 100% at each optimal pH

The effect of pH on the enzyme activity of MBP-Lcc120-3 for ABTS and DMP at a final buffer concentration of 50 mM was measured in acetate-NaOH buffer (pH 4.0–6.0), citrate-NaOH buffer (pH 5.5–7.0), KPB (pH 6.5–8.0), and Tris–HCl buffer (pH 7.5–9.0). The maximum activity of the enzyme reaction for ABTS was 100% at pH 4.5, 46% at pH 7.0, and 18% at pH 8.0 (Fig. 3). On the other hand, the maximum activity for DMP was observed at pH 7.5, indicating sufficient activity at neutral and slightly alkaline conditions. This is a remarkable characteristic for Lcc120-3 because most known Lccs reported mainly in basidiomycetes are only active at acidic conditions (pH 4.0–5.0) and are inactive around neutral pH (Baldrian 2006).

#### Effect of temperature on enzyme stability

**Fig. 4:**
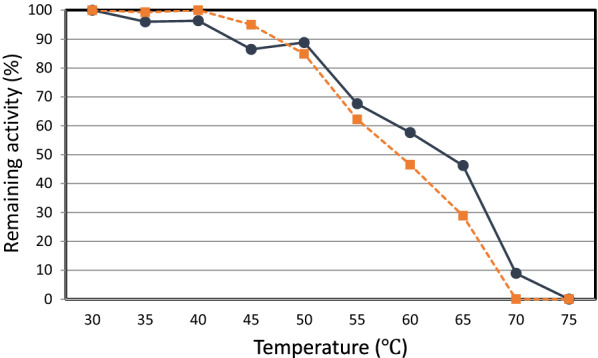
Thermal stability of MBP-Lcc120-3 and Lcc120-3. The thermal stability of the enzyme was measured by incubating the crude (MBP-Lcc) (dotted line) or purified enzyme (Lcc) (solid line) at each tested temperature for 30 min in 50 mM KPB (pH 7.0), followed by measuring the remaining activity for ABTS

The thermal stability of Lcc120-3 and MBP-Lcc120-3 was measured by incubating the purified or crude enzyme at each tested temperature for 30 min in 50 mM KPB (pH 7.0). Lcc120-3 was stable after incubation below 50 °C, and 47% and 9% of its original activity was maintained after incubation at 65 °C and 70 °C, respectively (Fig. 4), indicating sufficient thermal stability. The data showed that MBP-Lcc120-3 had slightly lower thermal stability than Lcc120-3.

#### Substrate specificity and kinetic parameters

The activity and kinetic parameters of purified Lcc120-3 were measured for various substrates (Table 4). The enzyme showed relatively high activity toward pyrogallol, *o*-catechol, DMP, and ABTS, which are well-known substrates of Lcc. Conversely, activity toward guaiacol and gallic acid was low compared with the other substrates. l-tyrosine, l-DOPA, bilirubin, syringaldazine, and iodide ions were not substrates of Lcc120-3. Because syringaldazine is an Lcc-specific compound, the lack of this activity in Lcc120-3 was characteristic of this enzyme (Baldrian 2006; Mano 2012), suggesting that Lcc120-3 is an Lcc-like enzyme and not a tyrosinase-, BOX-, and iodide oxidase-like enzyme. As summarized in Table 4, the specific activity of Lcc120-3 toward DMP (2.0 U/mg, *k*_cat_: ca. 2.3 s^−1^) was much lower than that of Lccs reported in basidiomycetes, for example, 165 U/mg (*k*_cat_: 185 s^−1^) of Lcc2 from *Hericium coralloides* (Itoh et al. 2016a), but was comparable with those ranging from 3 to 57 s^−1^ of bacteria such as *Bacillus* sp. CotAs (Mano 2012). The *K*_m_ and *V*_max_ values toward DMP calculated from a Lineweaver–Burk plot were 10.5 mM and 2.3 U/mg, respectively. Such a high *K*_m_ value for DMP has not been reported for known Lccs, suggesting that Lcc120-3 might possess a rather different substrate specificity from known Lccs. Notably, chimeric MBP-Lcc120-3 (MBP-Mic-mg-MCO) had fourfold higher activity for ABTS than the original MBP-Mic-MCO (Table 3). This means that the core region isolated from the metagenomes possesses a positive effect increasing Lcc activity.

**Table 4:** Substrate specificity and kinetic parameters of Lcc120-3

Substrate	Lcc120-3 (Mic-mg-MCO)	
	Activity (U/mg protein)	*K*_m_ (mM)*/V*_max_ (U/mg protein)/*k*_cat_ (s^−1^)
DMP	2.0	10.5/2.3/2.3
*o*-Guaiacol	0.05	–
*o*-Catechol	2.47	–
Pyrogallol	13.9	–
Gallic acid	0.64 POU	–
ABTS	6.63	–
Syringaldazine	N.D	–
Bilirubin	N.D	–
l-Tyrosine	N.D	–
l-DOPA (l-3,4-Dihydroxyphenylalanine)	N.D	–
Iodide	N.D	–

*N.D.* not detected

## Discussion

Eukaryote Lccs mainly isolated from basidiomycetes have been heterologously expressed in *Saccharomyces cerevisiae* (Hoshida et al. 2005)*,* the methylotrophic yeast *Pichia pastoris* (Hong et al. 2007), and the filamentous fungi *Aspergillus niger*, *Aspergillus oryzae* (Hoshida et al. 2005; Sigoillot et al. 2002), and *Trichoderma reesei* (Kiiskinen et al. 2004). However, it is difficult to sufficiently express the eukaryote Lcc genes in *E. coli* cells except in some special cases (Zelena et al. 2014; Ma et al. 2018). On the other hand, prokaryote MCOs such as from *Bacillus subtilis* (Enguita et al. 2003), *Bacillus licheniformis* (Koschorreck et al. 2008), and *Bacillus pumilus* (Reiss et al. 2011) have been overproduced in *E. coli*, especially *B. licheniformis* Lcc (Koschorreck et al. 2008). Based on this information, we focused on *Bacillus* sp. CotA genes, which are similar to the genes of the target MCO. Moreover, we adopted the MBP tag (di Guan et al. 1988) and *E. coli* chaperone system to support the soluble expression of *mco* genes in *E. coli*.

The S-GAM method we have developed includes the following steps (Itoh 2017): (1) Design of degenerate PCR primers (GAM primers) based on the conserved regions as near as possible to the N- and C-terminal regions in alignments constructed from the target gene; these primers are 25–30 bp in length and contain 15-bp regions that match the sequences of the known target enzyme gene. The 15-bp long regions are used to ligate the terminal regions with the expression vector using the In-Fusion method. (2) PCR using the GAM-primer set for various metagenomic samples. (3) Connection of the amplified amplicons with the linearized expression vector, and construction of *E*. *coli* libraries. (4) Screening of the libraries by simple enzyme activity or another high-throughput screening method. The obtained enzymes are all chimeric proteins with N- and C-terminal regions matching the known target enzyme. (5) If necessary, follow-up sequence analysis is performed. S-GAM is defined by steps 1 and 3, which enable omission of time-consuming subcloning and expression optimization procedures. To date, at least three enzyme genes including short-chain alcohol dehydrogenase (Itoh et al. 2014), zinc-dependent medium-chain alcohol dehydrogenase (Itoh et al. 2016b), and styrene monooxygenase genes have been isolated by this method and characterized. In this study, we applied this approach to bacterial MCO genes in the soil environment because bacterial MCOs represented by *Bacillus* sp. CotA (Lcc/BOX) have superior enzymatic properties and especially sufficient activity around neutral pH. However, we were unable to find suitable conserved regions matching the known bacterial MCO genes at the N- and C-terminal regions (Fig. 1). Thus, the isolation, expression, and detection of *mco*/*lcc* genes by the S-GAM technique using *E. coli* as a host cell is challenging. First, we modified this technique to select metagenomic samples from which the target genes can be amplified using normal degenerate primer sets and optimized the amplification conditions. Then, we used the GAM-primer set to amplify the core region of target genes from specific metagenomic samples. This modified technique functioned sufficiently well and the targeted *mco* genes were amplified by the GAM-primer set. Moreover, we expected that the chimeric MCOs/Lccs constructed possessing long flanking regions at the N- and C-termini might be inactive because they would be incompatible with the core regions isolated from the metagenomes. In fact, approximately 2000 clones constructed with the core genes and flanking regions of BsMCO were all inactive in *E. coli*. Accordingly, we changed the strategy from isolating diverse *mco* genes from metagenomes to optimizing or changing the N- and C-termini to match the specific core gene. Sequence analysis of major isolates indicated high similarity with the putative *mco* of *Micromonospora* sp. MP36 (ca. 70% amino acid identity) and that of *Comamonadaceae* (accession no. TY22925.1) (ca. 60% amino acid identity). Therefore, we substituted the N- and C-terminal regions of BsMCO (MBP-Bs-mg-MCO) with *Micromonospora* sp. MP36 MCO by using a different expression vector (pMAL-c5x-delMicMCO) to give MBP-Mic-mg-MCO.

As described in the Results, the chimeric enzyme (MBP-Mic-mg-MCO/Mic-mg-MCO) was active and demonstrated Lcc-specific characteristics, especially sufficient activity at neutral pH, a medium range of substrate specificity, and thermal stability. Interestingly, the constructed chimeric MBP-Lcc120-3 (MBP-Mic-mg-MCO) had much higher enzymatic activity than the original MBP-MicMCO (Table 3), although the effect of the MBP tag is not completely negligible. This suggested that using the S-GAM method to produce a chimeric enzyme can result in the generation of a superior enzyme as a protein engineering tool. Although the enzyme production level (0.09–0.16 U/mL-culture for ABTS) in *E. coli* was slightly lower than in a previous report using *E. coli* as a host, for example, 0.37 U/mL-culture for ABTS of *B. licheniformis* CotA (Lcc) (Koschorreck et al. 2008), further optimization of enzyme production should increase productivity. Chimeric Lcc120-3 (Mic-mg-MCO) was identified as a novel and unique Lcc in terms of its enzymatic properties, which could be applied as a biocatalyst in several reactions at neutral pH.

In this study, we were able to modify the S-GAM method to isolate a core enzyme gene from metagenomes and construct a functional chimeric enzyme by optimizing the N- and C-terminal flanking regions. This method would be a powerful alternative for traditional metagenomics for identifying enzyme genes as follows: (1) metagenomic DNA library construction using cosmids, BACs, or other vector systems, and then sequence-based screening using a DNA probe or PCR; (2) function- and activity-based screening of metagenomic DNA libraries; and (3) comprehensive sequencing of metagenomic DNA or DNA libraries to build an in-house database. In 2010, Ye et al. first reported a metagenome-derived novel Lcc by activity-based screening of a mangrove soil-metagenomic library. To date, some superior or unique Lccs have been isolated from marine microbe (Fang et al. 2011), sediment (Yang et al. 2018), soil (Ausec et al. 2017), and chemical plant sludge (Yue et al. 2017) metagenomes. Such data, including our present study, indicate that metagenomics is a powerful tool to isolate microbial *mco* genes from nature.

## Supplementary Information

**Additional file 1.** Nucleotide and amino acid sequences of constructed or isolated Lcc genes.

## Data Availability

The datasets generated during and/or analyzed during the current study are available from the corresponding author on reasonable request.

## Abbreviations

S-GAM: Screening of gene-specific amplicons from metagenomes
MCO: Multi-copper oxidase
BOX: Bilirubin oxidase
MBP: Maltose-binding protein
*E. coli*: *Escherichia coli*
Lcc: Laccase
PCR: Polymerase chain reaction
LB: Luria–Bertani
DMP: 2,6-Dimethoxyphenol
IPTG: Isopropyl-β-thiogalactopyranoside
ABTS: 2,2′-Azino-bis-(3-ethylthiazoline-6-sulphonate)
POU: Polyphenol oxidase unit
SDS-PAGE: Sodium dodecyl sulfate-polyacrylamide gel electrophoresis
l-DPOA: l-3,4-Dihydroxyphenylalanine

## References

[CR1] Amachi S, Muramatsu Y, Akiyama Y, Miyazaki K, Yoshiki S, Kamagata Y, Ban-nai T, Shinoyama H, Fuji T (2005). Isolation of iodide-oxidizing bacteria from iodide-rich natural gas brines and seawaters. Microbial Ecol.

[CR2] Aniszewski T, Lieberei R, Gulewicz K (2008). Research on catecholases, laccases and cresolases in plants. Recent progress and future needs. Acta Biol Cracov Bot.

[CR3] Ausec L, Berini F, Casciello C, Cretoiu MS, van Elsas JD, Marinelli F, Mandic-Mulec I (2017). The first acidobacterial laccase-like multicopper oxidase revealed by metagenomics shows high salt and thermo-tolerance. Appl Microbiol Biotecnol.

[CR4] Baldrian P (2006). Fungal laccases-occurrence and properties. FEMS Microbiol Rev.

[CR5] Brijwant K, Rigdon A, Vadlami PV (2010). Fungal laccases: production, function, and application in food processing. Enzyme Res.

[CR6] Camarero S, Cañas AI, Nousiainen P, Record E, Lomascolo A, Martínez MJ, Martínez AT (2008). *p*-Hydroxycinnamic acids as natural mediators for laccase oxidation of recalcitrant compounds. Environ Sci Technol.

[CR7] Chauhan PS, Goradia B, Saxena A (2017). Bacterial laccase: recent update on production, properties and industrial applications. 3 Biotech.

[CR8] Cracknell JA, McNamara TP, Lowe ED, Blanford CF (2011). Bilirubin oxidase from *Myrothecium verrucaria*: X-ray determination of the complete crystal structure and a rational surface modification for enhanced electrocatalytic O_2_ reduction. Dalton Trans.

[CR9] di Guan C, Li C, Riggs PD, Inouye H (1988). Vectors that facilitate the expression and purification of foreign peptides in *Escherichia coli* by fusion to maltose-binding protein. Gene.

[CR10] Díaz-Rodríguez A, Martínez-Montero L, Lavandera I, Gotor V, Gotor-Fernández V (2014). Laccase/2,2,6,6-tetramethylpiperidinoxyl radical (TEMPO): an efficient catalytic system for selective oxidations of primary hydroxy and amino groups in aqueous and biphasic media. Adv Synth Catal.

[CR11] Durand F, Kjaergaard CH, Suraniti E, Gounel S, Hadt RG, Solomon EI, Mano N (2012). Bilirubin oxidase from *Bacillus pumilus*: a promising enzyme for the elaboration of efficient cathodes in biofuel cells. Biosens Bioelectron.

[CR12] Dwivedi UN, Singh P, Pandey VP, Kumar A (2011). Structure-function relationship among bacterial, fungal and plant laccases. J Mol Catal B Enzym.

[CR13] Enguita FJ, Martins LO, Henriques AO, Carrondo MA (2003). Crystal structure of a bacterial endospore coat component. J Biol Chem.

[CR14] Fang Z, Li T, Wang Q, Zhang X, Peng H, Fang W, Hong Y, Ge H, Xiao Y (2011). A bacterial laccase from marine microbial metagenome exhibiting chloride tolerance and dye decolorization ability. Appl Microbiol Biotechnol.

[CR15] Hong Y, Zhou H, Tu X, Li J, Xiao Y (2007). Cloning of a laccase gene from a novel basidiomycete *Trametes* sp. 420 and its heterologous expression in *Pichia pastoris*. Curr Microbiol.

[CR16] Hoshida H, Fujita T, Murata K, Kubo K, Akada R (2005). Copper-dependent production of a *Pycnoporus coccineus* extracellular laccase in *Aspergillus oryzae* and *Saccharomyces cerevisiae*. Biosci Biotechnol Biochem.

[CR17] Hullo MF, Moszer I, Danchin A, Martin-Verstraete I (2001). CotA of *Bacillus subtilis* is a copper-dependent laccase. J Bacteriol.

[CR18] Itoh N, Matsuda T (2017). Metagenomics for improved biocatalysis. Future directions in biocatalysis.

[CR19] Itoh N, Kariya S, Kurokawa J (2014) Efficient PCR-based amplification of diverse alcohol dehydrogenase genes from metagenomes for improving biocatalysis: screening of gene-specific amplicons from metagenomes. Appl Environ Microbiol 80:6280–6289/Erratum (2016) 82:61 10.1128/AEM.01529-14 PMC4178635 25085492

[CR20] Itoh N, Kazama M, Takeuchi N, Isotani K, Kurokawa J (2016). Characterization and cloning of laccase from *Hericium coralloides* NBRC 7716 suitable for production of epitheaflagallin 3-*O*-gallate. Enzyme Microb Technol.

[CR21] Itoh N, Takagi S, Miki A, Kurokawa J (2016). Gene-specific amplicons from metagenomes as an alternative to directed evolution for enzyme screening: a case study using phenylacetaldehyde reductases. FEBS Open Bio.

[CR22] Itoh N, Kurokawa J, Isogai Y, Ogasawara M, Matsunaga T, Okubo T (2017). Functional characterization of epitheaflagallin 3-*O*-gallate generated in laccase-treated green tea extracts in the presence of gallic acid. J Agric Food Chem.

[CR23] Kiiskinen LL, Kruus K, Bailey M, Ylösmäki E, Siika-aho M, Saloheimo M (2004). Expression of *Melanocarpus albomyces* laccase in *Trichoderma reesei* and characterization of the purified enzyme. Microbiology.

[CR24] Koschorreck K, Richter SM, Ene AB, Roduner E, Schmid RD, Urlacher VB (2008). Cloning and characterization of a new laccase from *Bacillus licheniformis* catalyzing dimerization of phenolic acids. Appl Microbiol Biotecnol.

[CR25] Laemmli UK (1970). Cleavage of structural proteins during the assembly of the head of bacteriophage T4. Nature.

[CR26] Ma S, Liu N, Jia H, Dai D, Zang J, Cao Z, Dong J (2018). Expression, purification, and characterization of a novel laccase from *Setosphaeria turcica* in *Eschericha coli*. J Basic Microbiol.

[CR27] Mano N (2012). Features and application of bilirubin oxidase. Appl Microbiol Biotecnol.

[CR28] Mogharabi M, Faramarzi MA (2014). Laccase and laccase-mediated systems in the synthesis of organic compounds. Adv Synth Catal.

[CR29] Murao S, Tanaka N (1981). A new enzyme “bilirubin oxidase” produced by *Myrothecium verrucaria* MT-1. Agric Biol Chem.

[CR30] Reiss R, Ihssen J, Thöny-Meyer L (2011). *Bacillus pumilus* laccase: a heat stable enzyme with a wide substrate spectrum. BMC Biotechnol.

[CR31] Riva S (2006). Laccases: blue enzymes for green chemistry. Trends Biotechnol.

[CR32] Sakurai T, Kataoka K (2007). Basic and applied features of multicopper oxidases, CueO, bilirubin oxidases, and laccase. Chem Rec.

[CR33] Sambrook J, Russell WD (2001). Molecular cloning, a laboratory manual.

[CR34] Shraddha A, Shekher R, Sehgal S, Kamthania M, Kumar A (2011). Laccase: microbial sources, production, purification, and potential biotechnological applications. Enzyme Res.

[CR35] Sigoillot C, Record E, Belle V, Robert JL, Levasseur A, Punt PJ, van den Hondel CAMJJ, Fournel A, Sigoillot JC, Asther M (2002). Natural and recombinant fungal laccases for paper pulp bleaching. Appl Microbiol Biotechnol.

[CR36] Tanaka and Murao (1982). A new enzyme bilirubin oxidase produced by *Myrothecium verrucaria* MT-1. Agric Biol Chem.

[CR37] Yang Q, Zhang M, Zhang M, Wang C, Liu Y, Fan X, Li H (2018). Characterization of a novel, cold-adapted, and thermostable laccase-like enzyme with high tolerance for organic solvents and salt and potent dye decolorization ability, derived from a marine metagenomic library. Front Microbiol.

[CR38] Ye M, Li G, Liang WQ, Liu YH (2010). Molecular cloning and characterization of a novel metagenome-derived multicopper oxidase with alkaline activity and high soluble expression. Appl Microbiol Biotechnol.

[CR39] Yue Q, Yang Y, Zhao J, Zhang L, Xu L, Chu X, Liu X, Tian J, Wu N (2017). Identification of bacterial laccase *cueO* mutation from the metagenome of chemical plant sludge. Bioresour Bioprocess.

[CR40] Zelena K, Eisele N, Berger RG (2014). *Escherichia coli* as a production host for novel enzymes from Basidiomycota. Biotechnol Adv.

