# Supplementary material for: Construction and characterization of a functional chimeric laccase from metagenomes suitable as a biocatalyst
Source: AMB Express. 2021 Jun 19;11:90. doi: 10.1186/s13568-021-01248-y (PMC8214651; doi:10.1186/s13568-021-01248-y)
Supplement: Supplementary file 1 — Additional file 1. Nucleotide and amino acid sequences of constructed or isolated Lcc genes. [file 13568_2021_1248_MOESM1_ESM.pdf]

AMB Express

Construction and characterization of a functional chimeric laccase from metagenomes suitable as a biocatalyst

Nobuya Itoh*, Yuya Hayashi, Serina Honda, Yuna Yamamoto, Daichi Tanaka and Hiroshi Toda

Biotechnology and Pharmaceutical Engineering Research Center and Department of Biotechnology, Toyama Prefectural University, 5180 Kurokawa, Imizu, Toyama 939-0398, Japan

*Corresponding author, Nobuya Itoh, E-mail: nbito@pu-toyama.ac.jp/ itohnobuya123@gmail.com, TEL: +81 766 56 7500, FAX: +81 766 56 2498

Additional file 1 Nucleotide and amino acid sequences of constructed or isolated Lcc genes.

>s120-3 Mic-Mg-MCO
ATGAGCATTCAACCAGTGGCCCATCCACCAGCGCGTCCACCACGTCGTCGCCGTCT
CGCCCGTTGGCTGGGCGGTTTTCTGGCGGTTGCGCTGCTGGTTCCAGCCGCCGCC
GCCGGCGTTCTCGCGCTGCTGTGGGCCAATGCGGTTACCAACACGGCCGGTCGCA
CCGTTTTCGTTCGCCCGCTGGCCATTCCACCACTGGCGACCAGTCGCGTTGAAGAC
GGTCGCCGCGTTTTTGACCTCCGCGCGATCGAGGGCGTGAGTGATTTTGGTGTGG
GCGATCGCAAAACGCTGACTTGGGGTTATAACGGCTCGTACCTGGGACCGACGCT
GCGTGCCACGCGTGGCGAGCACGTGCAGGTGAACGTCACCAACGAGGTGGGCGA
GACCACGACCACGCACTGGCACGGGCACCACCTGCCGGCGGAGATGGACGGCGG
CCCGCACCAGCCGATCGAGCCCGGGCAGACCTGGAGTCCGCACTGGACGATCGAC
CAGCCGGCCGCCACCACCTGGTACCACCCGCACCTGCACGGCGCCACCGCCGCGC
ACGTGTACCGCGGCCTGGCCGGCATGTTCATCCTCGACGACGCCAACAGCCTCGC
GCTGGACCTGCCGTCCACGTACGGCGTCGACGACATCCCGCTGATCGTGCAGGAC
CGCGCGTTCGACTCCGCCGGCCGGGTGGTGGACCGGGCCCCGATGTTCAGCCCGG
TCGGGTCGCTGGGCGACACCGTGCTGGCCAACGGCACCGTGGGCGGCTACCTCGA
GGTCACCACCGAGCTGGTGCGGCTGCGGCTGCTCAACGGCTCCAACGCACGGGTC
TACGACTTCGGGTTCTCCGACGGGCGGGAGTTCTCGCTCATCGCCACCGACGGCG
GCCTGCTGCCCGCGCCGCACGTGACCGACCACGTCCAGCTGTCGCCGGGCGAGCG

GGCGGAGATCGTGGTGGCGATGCGGCCGGGCGAGGACGTGGTGCTGCGTAGCCG
GGACTCGGACCTCGGCGCCGGCTTCCTCGAACGGTTCTCCGGCGGGGACGACTCC
TTCGACGTCGTGCAGCTGCGCGCCGCCGACCGGTTGGCACCCTCCCCGCCGCTGC
CGCAGCGGCTGGCGCCGCCGCCGGACCTCGACCCGACGGACGCGCGGGTCACCC
GCACGTTCCGGATGGGGGACATGAACATCAACGGGACGTCGATGGATATGGGGCG
CATCGACGAGGTGGTGGAGGTCGACAGCACCGAGATCTGGGAGGTCTCCAACGCC
GACGGCGTTCCGCACAGCTTCCACGTCCACGACGTCCAGTTCCGCGTCGTGGCGG
TGGACGGCCGGCCGCCCGGGCCGGAGCTGTCGGGGTGGAAGGACACGGTGTACG
TCGCACCGGGTGGCACCGTCCGGCTGGTGCTGCGGTTCACCGACTACACCGACCC
GGTCTCGCCGTATATGTACCACTGTCACATGCTGCGCCATGAGGATCGCGGCATGA
TGGGTCAGTTCGTTGTGGTTCGCAAAGGCCAGCGTCCGGGTCGTCCACCAGCCGC
CGAGCATGATCATCAAGGCAGTGGCGGTGGTCTCCTCCCGGATCGTCCACCGGCC
GGTGCCGGCACCAGTGTTCGTCGCTAA

MSIQPVAHPPARPPRRRRLARWLGGFLAVALLVPAAAAGVLALLWANAVTNTAGRTV
FVRPLAIPPLATSRVEDGRRVFDLRAIEGVSDFGVGDRKTLTWGYNGSYLGPTLRAT
RGEHVQVNVTNEVGETTTTHWHGHHLPAEMDGGPHQPIEPGQTWSPHWTIDQPA
ATTWYHPHLHGATAAHVYRGLAGMFILDDANSLALDLPSTYGVDDIPLIVQDRAFD
SAGRVVDRAPMFSPVGSLGDTVLANGTVGGYLEVTTELVRLRLLNGSNARVYDFGF
SDGREFSLIATDGGLLPAPHVTDHVQLSPGERAEIVVAMRPGEDVVLRSRDSDLGAG
FLERFSGGDDSFDVVQLRAADRLAPSPPLPQRLAPPPDLDPTDARVTRTFRMGDMN
INGTSMDMGRIDEVVEVDSTEIWEVSNADGVPHSFHVHDVQFRVVAVDGRPPGPEL
SGWKDTVYVAPGGTVRLVLRFTDYTDPVSPYMYHCHMLRHEDRGMMGQFVVVRK
GQRPGRPPAAEHDHQGSGGGLLPDRPPAGAGTSVRR

>s120-3 MBP-Mic-Mg-MCO
MKIEEGKLVIWINGDKGYNGLAEVGKKFEKDTGIKVTVEHPDKLEEKFPQVAATGD
GPDIIFWAHDRFGGYAQSGLLAEITPDKAFQDKLYPFTWDAVRYNGKLIAYPIAVEAL
SLIYNKDLLPNPPKTWEEIPALDKELKAKGKSALMFNLQEPYFTWPLIAADGGYAF
KYENGKYDIKDVGVDNAGAKAGLTFLVDLIKNKHMNADTDYSIAEAAFNKGETAM
TINGPWAWSNIDTSKVNYGVTVLPTFKGQPSKPFVGVLSAGINAASPNKELAKEFLE
NYLLTDEGLEAVNKDKPLGAVALKSYEEELVKDPRIAATMENAQKGEIMPNIPQMS
AFWYAVRTAVINAASGRQTVDEALKDAQTNSSSNNNNNNNNNNLGIEGRISHMSIQ
PVAHPPARPPRRRRLARWLGGFLAVALLVPAAAAGVLALLWANAVTNTAGRTVFVRP
LAIPPLATSRVEDGRRVFDLRAIEGVSDFGVGDRKTLTWGYNGSYLGPTLRATRGEH
VQVNVTNEVGETTTTHWHGHHLPAEMDGGPHQPIEPGQTWSPHWTIDQPAATTW

YHPHLHGATAAHVYRGLAGMFILDDANSLALDLPSTYGVDDIPLIVQDRAFDSAGRV
VDRAPMFSPVGSLGDTVLANGTVGGYLEVTTELVRLRLLNGSNARVYDFGFSDGRE
FSLIATDGGLLPAPHVTDHVQLSPGERAEIVVAMRPGEDVVLRSRDSDLGAGFLERF
SGGDDSFDVVQLRAADRLAPSPPLPQRLAPPPDLDPTDARVTRTFRMGDMNINGTS
MDMGRIDEVVEVDSTEIWEVSNADGVPHSFHVHDVQFRVVAVDGRPPGPELSGWK
DTVYVAPGGTVRLVLRFTDYTDPVSPYMYHCHMLRHEDRGMMGQFVVVRKGQRP
GRPPAAEHDHQGSGGGLLPDRPPAGAGTSVRR

▭ : FactorXa cleavage site

>Mic-MCO

MiCMCO（for E. coli）

ATGAGCATTCAACCAGTGGCCCATCCACCAGCGCGTCCACCACGTCGTCGCCGTCT
CGCCCGTTGGCTGGGCGGTTTTCTGGCGGTTGCGCTGCTGGTTCCAGCCGCCGCC
GCCGGCGTTCTCGCGCTGCTGTGGGCCAATGCGGTTACCAACACGGCCGGTCGCA
CCGTTTTCGTTCGCCCGCTGGCCATTCCACCACTGGCGACCAGTCGCGTTGAAGAC
GGTCGCCGCGTTTTTGACCTCCGCGCGATCGAGGGCGTGAGTGATTTTGGTGTGG
GCGATCGCAAAACGCTGACGTGGGGCTTTAATGGCAGTTATCTCGGTCCGACCCTC
CGTACGAAACGTGGCGAGCAAGTGCAGATCAACGTGAGCAACGAGCTGGGTGAAA
CGACGACCGTTCATTGGCATGGCCAGCATCTGCCAGCCGCGATGGATGGCGGTCC
ACACCAGATGGTTGAACCGGGCGAGACGTGGAGCCCGCATTGGCAAATCGATCAG
CCAGCGGCGACCACGTGGTATCATCCACATCTGCATGGCAGTACCGCGGCGCATGT
TCATCGCGGTCTGGCGGGCATGTTCATTCTGGATGACGATCGCACCGCCACGCTCG
GTCTGCCGAGCATGTATGGTGTGGATGACGTGCCACTGATCGTGCAAGATCGCAGC
TTTGACGCCGATGGTCAGTTCCACGACGGCGATCCGACCTTTAGCCCGGTGGGCTT
TCTGGGCGATACGATTCTGACCAATGGCACCATCGGTGCCTACCATGATGTTACCA
CCGAACTCGTGCGCCTCCGTATTCTGAACGGCAGCAATGCGCGTGTTTACGACTTT
GGTATGCATGATGACCGCGATTTCTTTCTGATCGGTACGGATGGTGGTCTGCTCCC
AGCCCCACATGCCACCAATCGTGTTCGTCTGAGTCCGGGCGAACGTGCCGAAATT
GTGGTTCGTGTGCGTGCCGGCGAACATGCGGTTCTGCGCAGTTACCCGCCGGAAC
TGGGTACGGATTTCTGGAACGAACGCTTTGCCGGCGGTGACGACACGTTTGATGT
GCTGCAACTGCGCGCGGCGGATAGTCTGACGAGTAGTCCGGCGCCACCAGAACGT
CTGGCCGATGCGCCGGATCTGGACGCGGCGGATGCGCGTAAAACCCGTACGTTCC
GTCTGGGTGGCAGTAACATCAACGGCAAGAAGATGGACATGAGCCGCATCGATGA
AGCCGTTGAGGTGGATACGACCGAAATCTGGGAAGTGACCAATCCGGGCAATACC
CCGCACAATTTCCACGTGCATGACGTGCAATTCGCCGTGCTCGAAGTTGGTGGTCG
CCGTCCGGGTCCGGAACTGAGTGGCTGGAAGGATACCATCTATGTTGCGCCGGGC

ACCACCGCGCGTCTGATTCTGCGTTTCAGCGACTATGCCGATCCGGACGTTCCGTA
CATGTTCCACTGCCATGTGCTGCGCCATGAGGATCGCGGCATGATGGGTCAGTTCG
TTGTGGTTCGCAAAGGCCAGCGTCCGGGTCGTCCACCAGCCGCCGAGCATGATCA
TCAAGGCAGTGGCGGTGGTCTCCTCCCGGATCGTCCACCGGCCGGTGCCGGCACC
AGTGTTCGTCGCTAA

MSIQPVAHPPARPPRRRRLARWLGGFLAVALLVPAAAAGVLALLWANAVTNTAGRTV
FVRPLAIPPLATSRVEDGRRVFDLRAIEGVSDFGVGDRKTLTWGFNGSYLGPTLRTK
RGEQVQINVSNELGETTTVHWHGQHLPAAMDGGPHQMVEPGETWSPHWQIDQPA
ATTWYHPHLHGSTAAHVHRGLAGMFILDDDRTATLGLPSMYGVDDVPLIVQDRSFD
ADGQFHDGDPTFSPVGFLGDTILTNGTIGAYHDVTTELVRLRILNGSNARVYDFGMH
DDRDFFLIGTDGGLLPAPHATNRVRLSPGERAEIVVRVRAGEHAVLRSYPPELGTDF
WNERFAGGDDTFDVLQLRAADSLTSSPAPPERLADAPDLDAADARKTRTFRLGGSN
INGKKMDMSRIDEAVEVDTTEIWEVTNPGNTPHNFHVHDVQFAVLEVGGRRPGPE
LSGWKDTIYVAPGTTARLILRFSDYADPDVPYMFHCHVLRHEDRGMMGQFVVVRK
GQRPGRPPAAEHDHQGSGGGLLPDRPPAGAGTSVRR

>MBP
ATGAAAATCGAAGAAGGTAAACTGGTAATCTGGATTAACGGCGATAAAGGCTATAA
CGGTCTCGCTGAAGTCGGTAAGAAATTCGAGAAAGATACCGGAATTAAAGTCACCG
TTGAGCATCCGGATAAACTGGAAGAGAAATTCCCACAGGTTGCGGCAACTGGCGAT
GGCCCTGACATTATCTTCTGGGCACACGACCGCTTTGGTGGCTACGCTCAATCTGG
CCTGTTGGCTGAAATCACCCCGGACAAAGCGTTCCAGGACAAGCTGTATCCGTTTA
CCTGGGATGCCGTACGTTACAACGGCAAGCTGATTGCTTACCCGATCGCTGTTGAA
GCGTTATCGCTGATTTATAACAAAGATCTGCTGCCGAACCCGCCAAAAACCTGGGA
AGAGATCCCGGCGCTGGATAAAGAACTGAAAGCGAAAGGTAAGAGCGCGCTGATG
TTCAACCTGCAAGAACCGTACTTCACCTGGCCGCTGATTGCTGCTGACGGGGGTTA
TGCGTTCAAGTATGAAAACGGCAAGTACGACATTAAAGACGTGGGCGTGGATAACG
CTGGCGCGAAAGCGGGTCTGACCTTCCTGGTTGACCTGATTAAAAACAAACACATG
AATGCAGACACCGATTACTCCATCGCAGAAGCTGCCTTTAATAAAGGCGAAACAGC
GATGACCATCAACGGCCCGTGGGCATGGTCCAACATCGACACCAGCAAAGTGAATT
ATGGTGTAACGGTACTGCCGACCTTCAAGGGTCAACCATCCAAACCGTTCGTTGGC
GTGCTGAGCGCAGGTATTAACGCCGCCAGTCCGAACAAAGAGCTGGCAAAAGAGT
TCCTCGAAAACTATCTGCTGACTGATGAAGGTCTGGAAGCGGTTAATAAAGACAAA
CCGCTGGGTGCCGTAGCGCTGAAGTCTTACGAGGAAGAGTTGGTGAAAGATCCGC

GTATTGCCGCCACTATGGAAAACGCCCAGAAAGGTGAAATCATGCCGAACATCCCG
CAGATGTCCGCTTTCTGGTATGCCGTGCGTACTGCGGTGATCAACGCCGCCAGCGG
TCGTCAGACTGTCGATGAAGCCCTGAAAGACGCGCAGACTAATTCGAGCTCGAAC
AACAACAACAATAACAATAACAACAACCTCGGGATCGAGGGAAGGATTTCACAT

MKIEEGKLVIWINGDKGYNGLAEVGKKFEKDTGIKVTVEHPDKLEEKFPQVAATGD
GPDIIFWAHDRFGGYAQSGLLAEITPDKAFQDKLYPFTWDAVRYNGKLIAYPIAVEAL
SLIYNKDLLPNPPKTWEEIPALDKELKAKGKSALMFNLQEPYFTWPLIAADGGYAF
KYENGKYDIKDVGVDNAGAKAGLTFLVDLIKNKHMNADTDYSIAEAAFNKGETAM
TINGPWAWSNIDTSKVNYGVTVLPTFKGQPSKPFVGVLSAGINAASPNKELAKEFLE
NYLLTDEGLEAVNKDKPLGAVALKSYEEELVKDPRIAATMENAQKGEIMPNIPQMS
AFWYAVRTAVINAASGRQTVDEALKDAQTNSSSNNNNNNNNNNLGIEGRISH

>s120-2
ACTTGGGGTTATAACGGTTCGTACCTGGGACCGACGCTGCGTGCCACGCGTGGCG
AGCACGTGCAGGTGAACGTCACCAACGAGGTGGGCGAGACCACGACCACGCACTG
GCACGGGCACCACCTGCCGGCGGAGATGGACGGCGGCCCGCACCAGCCGATCGA
GCCCGGGCAGACCTGGAGTCCGCACTGGACGATCGACCAGCCGGCCGCCACCACC
TGGTACCACCCGCACCTGCACGGCGCCACCGCCGCGCACGTGTACCGCGGCCTGG
CCGGCATGTTCATCCTCGACGACGCCAACAGCCTCGCGCTGGACCTGCCGTCCAC
GTACGGCGTCGACGACATCCCGCTGATCGTGCAGGACCGCGCGTTCGACTCCGCC
GGCCGGGTGGTGGACCGGGCCCCGATGTTCAGCCCGGTCGGGTCGCTGGGCGAC
ACCGTGCTGGCCAACGGCACCGTGGGCGGCTACCTCGAGGTCACCACCGAGCTGG
TGCGGCTGCGGCTGCTCAACGGCTCCAACGCACGGGTCTACGACTTCGGGTTCTC
CGACGGGCGGGAGTTCTCGCTCATCGCCACCGACGGCGGCCTGCTGCCCGCGCCG
CACGTGACCGACCACGTCCAGCTGTCGCCGGGCGAGCGGGCGGAGATCGTGGTG
GCGATGCGGCCGGGCGAGGACGTGGTGCTGCGTAGCCGGGACCCGGACCTCGGC
GCCGGCTTCCTCGAACGGTTCTCCGGCGGGGACGACTCCTTCGACGTCGTGCAGC
TGCGCGCCGCCGACCGGTTGGCACCCTCCCCGCCGCTGCCGCAGCGGCTGGCGCC
GCCGCCGGACCTCGACCCGACGGACGCGCGGGTCACCCGCACGTTCCGGATGGG
GGACATGAACATCAACGGGGCGTCGATGGATATGGGGCGCATCGACGAGGTGGTG
GAGGTCGACAGCACCGAGATCTGGGAGGTCTCCAACGCCGACGGCGTTCCGCACA
GCTTCCACGTCCACGACGTCCAGTTCCGCGTCGTGGCGGTGGACGGCCGGCCGCC
CGGGCCGGAGCTGTCGGGGTGGAAGGACACGGTGTACGTCGCACCGGGTGGCAC

CGTCCGGCTGGTGCTGCGGTTCACCGACTACACCGACCCGGACTCGCCGTATATGT
ACCACTGCCACTT

TWGYNGSYLGPTLRATRGEHVQVNVTNEVGETTTTHWHGHHLPAEMDGGPHQPI
EPGQTWSPHWTIDQPAATTWYHPHLHGATAAHVYRGLAGMFILDDANSLALDLPST
YGVDDIPLIVQDRAFDSAGRVVDRAPMFSPVGSLGDTVLANGTVGGYLEVTTELVRL
RLLNGSNARVYDFGFSDGREFSLIATDGGLLPAPHVTDHVQLSPGERAEIVVAMRPG
EDVVLRSRDPDLGAGFLERFSGGDDSFDVVQLRAADRLAPSPPLPQRLAPPPDLDPT
DARVTRTFRMGDMNINGASMDMGRIDEVVEVDSTEIWEVSNADGVPHSFHVHDVQ
FRVVAVDGRPPGPELSGWKDTVYVAPGGTVRLVLRFTDYTDPDSPYMYHCH

>s120-3
ACTTGGGGTTATAACGGCTCGTACCTGGGACCGACGCTGCGTGCCACGCGTGGCG
AGCACGTGCAGGTGAACGTCACCAACGAGGTGGGCGAGACCACGACCACGCACTG
GCACGGGCACCACCTGCCGGCGGAGATGGACGGCGGCCCGCACCAGCCGATCGA
GCCCGGGCAGACCTGGAGTCCGCACTGGACGATCGACCAGCCGGCCGCCACCACC
TGGTACCACCCGCACCTGCACGGCGCCACCGCCGCGCACGTGTACCGCGGCCTGG
CCGGCATGTTCATCCTCGACGACGCCAACAGCCTCGCGCTGGACCTGCCGTCCAC
GTACGGCGTCGACGACATCCCGCTGATCGTGCAGGACCGCGCGTTCGACTCCGCC
GGCCGGGTGGTGGACCGGGCCCCGATGTTCAGCCCGGTCGGGTCGCTGGGCGAC
ACCGTGCTGGCCAACGGCACCGTGGGCGGCTACCTCGAGGTCACCACCGAGCTGG
TGCGGCTGCGGCTGCTCAACGGCTCCAACGCACGGGTCTACGACTTCGGGTTCTC
CGACGGGCGGGAGTTCTCGCTCATCGCCACCGACGGCGGCCTGCTGCCCGCGCCG
CACGTGACCGACCACGTCCAGCTGTCGCCGGGCGAGCGGGCGGAGATCGTGGTG
GCGATGCGGCCGGGCGAGGACGTGGTGCTGCGTAGCCGGGACTCGGACCTCGGC
GCCGGCTTCCTCGAACGGTTCTCCGGCGGGGACGACTCCTTCGACGTCGTGCAGC
TGCGCGCCGCCGACCGGTTGGCACCCTCCCCGCCGCTGCCGCAGCGGCTGGCGCC
GCCGCCGGACCTCGACCCGACGGACGCGCGGGTCACCCGCACGTTCCGGATGGG
GGACATGAACATCAACGGGACGTCGATGGATATGGGGCGCATCGACGAGGTGGTG
GAGGTCGACAGCACCGAGATCTGGGAGGTCTCCAACGCCGACGGCGTTCCGCACA
GCTTCCACGTCCACGACGTCCAGTTCCGCGTCGTGGCGGTGGACGGCCGGCCGCC
CGGGCCGGAGCTGTCGGGGTGGAAGGACACGGTGTACGTCGCACCGGGTGGCAC
CGTCCGGCTGGTGCTGCGGTTCACCGACTACACCGACCCGGTCTCGCCGTATATGT
ACCACTGTCACAT

TWGYNGSYLGPTLRATRGEHVQVNVTNEVGETTTTHWHGHHLPAEMDGGPHQPI
EPGQTWSPHWTIDQPAATTWYHPHLHGATAAHVYRGLAGMFILDDANSLALDLPST
YGVDDIPLIVQDRAFDSAGRVVDRAPMFSPVGSLGDTVLANGTVGGYLEVTTELVRL
RLLNGSNARVYDFGFSDGREFSLIATDGGLLPAPHVTDHVQLSPGERAEIVVAMRPG
EDVVLRSRDSDLGAGFLERFSGGDDSFDVVQLRAADRLAPSPPLPQRLAPPPDLDPT
DARVTRTFRMGDMNINGTSMDMGRIDEVVEVDSTEIWEVSNADGVPHSFHVHDVQ
FRVVAVDGRPPGPELSGWKDTVYVAPGGTVRLVLRFTDYTDPVSPYMYHCH

>s120-4
ACGTGGGGTTATAATGGCTCGTACCTGGGACCGACGCTGCGTGCCACGCGTGGCG
AGCACGTGCAGGTGAACGTCACCAACGAGGTGGGCGAGACCACGACCACGCACTG
GCACGGGCACCACCTGCCGGCGGAGATGGACGGCGGCCCGCACCAGCCGATCGA
GCCCGGGCAGACCTGGAGTCCGCACTGGACGATCGACCAGCCGGCCGCCACCACC
TGGTACCACCCGCACCTGCACGGCGCCACCGCCGCGCACGTGTACCGCGGCCTGG
CCGGCATGTTCATCCTCGACGACGCCAACAGCCTCGCGCTGGACCTGCCGTCCAC
GTACGGCGTCGACGACATCCCGCTGATCGTGCAGGACCGCGCGTTCGACTCCGCC
GGCCGGGTGGTGGACCGGGCCCCGATGTTCAGCCCGGTCGGGTCGCTGGGCGAC
ACCGTGCTGGCCAACGGCACCGTGGGCGGCTACCTCGAGGTCACCACCGAGCTGG
TGCGGCTGCGGCTGCTCAACGGCTCCAACGCACGGGTCTACGACTTCGGGTTCTC
CGACGGGCGGGAGTTCTCGCTCATCGCCACCGACGGCGGCCTGCTGCCCGCGCCG
CACGTGACCGACCACGTCCAGCTGTCGCCGGGCGAGCGGGCGGAGATCGTGGTG
GCGATGCGGCCGGGCGAGGACGTGGTGCTGCGTAGCCGGGACCCGGACCTCGGC
GCCGGCTTCCTCGAACGGTTCTCCGGCGGGGACGACTCCTTCGACGTCGTGCAGC
TGCGCGCCGCCGACCGGTTGGCACCCTCCCCGCCGCTGCCGCAGCGGCTGGCGCC
GCCGCCGGACCTCGACCCGACGGACGCGCGGGTCACCCGCACGTTCCGGATGGG
GGACATGAACATCAACGGGACGTCGATGGATATGGGGCGCATCGACGAGGTGGTG
GAGGTCGACAGCACCGAGATCTGGGAGGTCTCCAACGCCGACGGCGTTCCGCACA
GCTTCCACGTCCACGACGTCCAGTTCCGCGTCGTGGCGGTGGACGGCCGGCCGCC
CGGGCCGGAGCTGTCGGGGTGGAAGGACACGGTGTACGTCGCACCGGGTGGCAC
CGTCCGGCTGGTGCTGCGGTTCACCGACTACACCGACCCGGACTCGCCGTTTATGT
ACCATTGTCACTT

TWGYNGSYLGPTLRATRGEHVQVNVTNEVGETTTTHWHGHHLPAEMDGGPHQPI
EPGQTWSPHWTIDQPAATTWYHPHLHGATAAHVYRGLAGMFILDDANSLALDLPST
YGVDDIPLIVQDRAFDSAGRVVDRAPMFSPVGSLGDTVLANGTVGGYLEVTTELVRL
RLLNGSNARVYDFGFSDGREFSLIATDGGLLPAPHVTDHVQLSPGERAEIVVAMRPG

EDVVLRSRDPDLGAGFLERFSGGDDSFDVVQLRAADRLAPSPPLPQRLAPPPDLDPT
DARVTRTFRMGDMNINGTSMDMGRIDEVVEVDSTEIWEVSNADGVPHSFHVHDVQ
FRVVAVDGRPPGPELSGWKDTVYVAPGGTVRLVLRFTDYTDPDSPFMYHCH

>s120-5
ACATGGGGTTACAATGGCACGTACCTGGGACCGACGCTGCGTGCCACGCGTGGCG
AGCACGTGCAGGTGAACGTCACCAACGAGGTGGGCGAGACCACGACCACGCACTG
GCACGGGCACCACCTGCCGGCGGAGATGGACGGCGGCCCGCACCAGCCGATCGA
GCCCGGGCAGACCTGGAGTCCGCACTGGACGATCGACCAGCCGGCCGCCACCACC
TGGTACCACCCGCACCTGCACGGCGCCACCGCCGCGCACGTGTACCGCGGCCTGG
CCGGCATGTTCATCCTCGACGACGCCAACAGCCTCGCGCTGGACCTGCCGTCCAC
GTACGGCGTCGACGACATCCCGCTGATCGTGCAGGACCGCGCGTTCGACTCCGCC
GGCCGGGCGGTGGACCGGGCCCCGATGTTCAGCCCGGTCGGGTCGCTGGGCGAC
ACCGTGCTGGCCAACGGCACCGTGGGCGGCTACCTCGAGGTCACCACCGAGCTGG
TGCGGCTGCGGCTGCTCAACGGCTCCAACGCACGGGTCTACGACTTCGGGTTCTC
CGACGGGCGGGAGTTCTCGCTCATCGCCACCGACGGCGGCCTGCTGCCCGCGCCG
CACGTGACCGACCACGTCCAGCTGTCGCCGGGCGAGCGGGCGGAGATCGTGGTG
GCGATGCGGCCGGGCGAGGACGTGGTGCTGCGTAGCCGGGACCCGGACCTCGGC
GCCGGCTTCCTCGAACGGTTCTCCGGCGGGGACGACTCCTTCGACGTCGTGCAGC
TGCGCGCCGCCGACCGGTTGGCACCCTCCCCGCCGCTGCCGCAGCGGCTGGCGCC
GCCGCGGGACCTCGACCCGACGGACGCGCGGGTCACCCGCGCGTTCCGGATGGG
GGACATGAACATCAACGGGGCGTCGATGGATATGGGGCGCATCGACGAGGTGGTG
GAGGTCGACAGCACCGAGATCTGGGAGGTCTCCAACGCCGACGGCGTTCCGCACA
GCTTCCACGTCCACGACGTCCAGTTCCGCGTCGTGGCGGTGGACGGCCGGCCGCC
CGGGCCGGAGCTGTCGGGGTGGAAGGACACGGTGTACGTCGCACCGGGTGGCAC
CGTCCGGCTGGTGCTGCGGTTCACCGACTACACCGACCCGGTCTCGCCGTTCATG
TATCACTGTCACAT

TWGYNGTYLGPTLRATRGEHVQVNVTNEVGETTTTHWHGHHLPAEMDGGPHQPI
EPGQTWSPHWTIDQPAATTWYHPHLHGATAAHVYRGLAGMFILDDANSLALDLPST
YGVDDIPLIVQDRAFDSAGRAVDRAPMFSPVGSLGDTVLANGTVGGYLEVTTELVRL
RLLNGSNARVYDFGFSDGREFSLIATDGGLLPAPHVTDHVQLSPGERAEIVVAMRPG
EDVVLRSRDPDLGAGFLERFSGGDDSFDVVQLRAADRLAPSPPLPQRLAPPRDLDP
TDARVTRAFRMGDMNINGASMDMGRIDEVVEVDSTEIWEVSNADGVPHSFHVHDV
QFRVVAVDGRPPGPELSGWKDTVYVAPGGTVRLVLRFTDYTDPVSPFMYHCH

>s121-1

ACCTGGGGATACAATGGAACGTACCTGGGACCGACGCTGCGTGCCACGCGTGGCG
AGCACGTGCAGGTGAACGTCACCAACGAGGTGGGCGAGACCACGACCACGCACTG
GCACGGGCACCACCTGCCGGCGGAGATGGACGGCGGCCCGCACCAGCCGATCGA
GCCCGGGCAGACCTGGAGTCCGCACTGGACGATCGACCAGCCGGCCGCCACCACC
TGGTACCACCCGCACCTGCACGGCGCCACCGCCGCGCACGTGTACCGCGGCCTGG
CCGGCATGTTCATCCTCGACGACGCCAACAGCCTCGCGCTGGACCTGCCGTCCAC
GTACGGCGTCGACGACATCCCGCTGATCGTGCAGGACCGCGCGTTCGACTCCGCC
GGCCGGGTGGTGGACCGGGCCCCGATGTTCAGCCCGGTCGGGTCGCTGGGCGAC
ACCGTGCTGGCCAACGGCACCGTGGGCGGCTACCTCGAGGTCACCACCGAGCTGG
TGCGGCTGCGGCTGCTCAACGGCTCCAACGCACGGGTCTACGACTTCGGGTTCTC
CGACGGGCGGGAGTTCTCGCTCATCGCCACCGACGGCGGCCTGCTGCCCGCGCCG
CACGTGACCGACCACGTCCAGCTGTCGCCGGGCGAGCGGGCGGAGATCGTGGTG
GCGATGCGGCCGGGCGAGGACGTGGTGCTGCGTAGCCGGGACCCGGACCTCGGC
GCCGGCTTCCTCGAACGGTTCTCCGGCGGGGACGACTCCTTCGACGTCGTGCAGC
TGCGCGCCGCCGACCGGTTGGCACCCTCCCCGCCGCTGCCGCAGCGGCTGGCGCC
GCCGCCGGACCTCGACCCGACGGACGCGCGGGTCACCCGCACGTTCCGGATGGG
GGACATGAACATCAACGGGGCGTCGATGGATATGGGGCGCATCGACGAGGTGGTG
GAGGTCGACAGCACCGAGATCTGGGAGGTCTCCAACGCCGACGGCGTTCCGCACA
GCTTCCACGTCCACGACGTCCAGTTCCGCGTCGTGGCGGTGGACGGCCGGCCGCC
CGGGCCGGAGCTGTCGGGGTGGAAGGACACGGTGTACGTCGCACCGGGTGGCAC
CGTCCGGCTGGTGCTGCGGTTCACCGACTACACCGACCCGGACTCGCCGTTTATGT
ATCACTGTCACTT

TWGYNGTYLGPTLRATRGEHVQVNVTNEVGETTTTHWHGHHLPAEMDGGPHQPI
EPGQTWSPHWTIDQPAATTWYHPHLHGATAAHVYRGLAGMFILDDANSLALDLPST
YGVDDIPLIVQDRAFDSAGRVVDRAPMFSPVGSLGDTVLANGTVGGYLEVTTELVRL
RLLNGSNARVYDFGFSDGREFSLIATDGGLLPAPHVTDHVQLSPGERAEIVVAMRPG
EDVVLRSRDPDLGAGFLERFSGGDDSFDVVQLRAADRLAPSPPLPQRLAPPPDLDPT
DARVTRTFRMGDMNINGASMDMGRIDEVVEVDSTEIWEVSNADGVPHSFHVHDVQ
FRVVAVDGRPPGPELSGWKDTVYVAPGGTVRLVLRFTDYTDPDSPFMYHCH

>s123-1

ACTTGGGGATATAATGGATCGTACCTGGGACCGACGCTGCGTGCCACGCGTGGCG
AGCACGTGCAGGTGAACGTCACCAACGAGGTGGGCGAGACCACGACCACGCACTG

GCACGGGCACCACCTGCCGGCGGAGATGGACGGCGGCCCGCACCAGCCGATCGA
GCCCGGGCAGACCTGGAGTCCGCACTGGACGATCGACCAGCCGGCCGCCACCACC
TGGTACCACCCGCACCTGCACGGCGCCACCGCCGCGCACGTGTACCGCGGCCTGG
CCGGCATGTTCATCCTCGACGACGCCAACAGCCTCGCGCTGGACCTGCCGTCCAC
GTACGGCGTCGACGACATCCCGCTGATCGTGCAGGACCGCGCGTTCGACTCCGCC
GGCCGGGTGGTGGACCGGGCCCCGATGTTCAGCCCGGTCGGGTCGCTGGGCGAC
ACCGTGCTGGCCAACGGCACCGTGGGCGGCTACCTCGAGGTCACCACCGAGCTGG
TGCGGCTGCGGCTGCTCAACGGCTCCAACGCACGGGTCTACGACTTCGGGTTCTC
CGACGGGCGGGAGTTCTCGCTCATCGCCACCGACGGCGGCCTGCTGCCCGCGCCG
CACGTGACCGACCACGTCCAGCTGTCGCCGGGCGAGCGGGCGGAGATCGTGGTG
GCGATGCGGCCGGGCGAGGACGTGGTGCTGCGTAGCCGGGACTCGGACCTCGGC
GCCGGCTTCCTCGAACGGTTCTCCGGCGGGGACGACTCCTTCGACGTCGTGCAGC
TGCGCGCCGCCGACCGGTTGGCACCCTCCCCGCCGCTGCCGCAGCGGCTGGCGCC
GCCGCCGGACCTCGACCCGACGGACGCGCGGGTCACCCGCACGTTCCGGATGGG
GGACATGAACATCAACGGGGCGTCGATGGATATGGGGCGCATCGACGAGGTGGTG
GAGGTCGACAGCACCGAGATCTGGGAGGTCTCCAACGCCGACGGCGTTCCGCACA
GCTTCCACGTCCACGACGTCCAGTTCCGCGTCGTGGCGGTGGACGGCCGGCCGCC
CGGGCCGGAGCTGTCGGGGTGGAAGGACACGGTGTACGTCGCACCGGGTGGCAC
CGTCCGGCTGGTGCTGCGGTTCACCGACTACACCGACCCGGACTCGCCGTATATGT
ACCACTGCCACAT

TWGYNGSYLGPTLRATRGEHVQVNVTNEVGETTTTHWHGHHLPAEMDGGPHQPI
EPGQTWSPHWTIDQPAATTWYHPHLHGATAAHVYRGLAGMFILDDANSLALDLPST
YGVDDIPLIVQDRAFDSAGRVVDRAPMFSPVGSLGDTVLANGTVGGYLEVTTELVRL
RLLNGSNARVYDFGFSDGREFSLIATDGGLLPAPHVTDHVQLSPGERAEIVVAMRPG
EDVVLRSRDSDLGAGFLERFSGGDDSFDVVQLRAADRLAPSPPLPQRLAPPPDLDPT
DARVTRTFRMGDMNINGASMDMGRIDEVVEVDSTEIWEVSNADGVPHSFHVHDVQ
FRVVAVDGRPPGPELSGWKDTVYVAPGGTVRLVLRFTDYTDPDSPYMYHCH

>s123-3
ACTTGGGGTTATAATGGAAGCTTCCTCGGCCCGACCCTGCGGCTGCACTCCGGCA
GCGAGGCGCGCGTGACCGTGCGCAACCGGCTGCCCGAGCCGACGACGGTGCACT
GGCACGGCCTGCTGGTGCCCGCGGCCGTGGACGGCGGCCCGCACAACGAGATCG
CGCCCGACGGCGGGATGTGGCAACCCGTGCTGCCGGTGCGCCAGCCGGCCGCCA
CCGCCTGGTACCACGCGCATCCCCACATGCGCACCGCGCAGCAGGTCTATGCCGG
GCTGGCCGGCATGCTGATCGTGACCGACGCCGAGGAGCAGGCGCTCGGCCTGCCA

TCGCGCTACGGGGTGGACGACCTGCCCCTGATCCTGCAGGACCGCTTCCTGGATG
GCAGCGGCCGCATGCTCTATCCGCGCGGGCCGATGACGCAGATGCACGGCGCCTT
CGGCAACACGCTGCTCGTCAACGGGGCGCCCGGGCCGCTGGCCCGCGTGCCGGC
CGCGCTGGTGCGGCTGCGGCTGCTGAACGCGGCCAACGCGCGCAGCTTCGACCTG
GCCTTCGCCCGACGGCCGCAGCTTTCCCAGTGGGATCGCCACGGGAAAGGCGGCC
TGCTGCGCGAGCCCGTGACGCGCACGCGCCTGCTTCTGGCGCCGGCCCAGCGGG
CCGAACTGCTGGTGGACTTCTCCGACGGCGCGCCCGCCATGCTGGGCACGCATGC
CGCCGGCATGCTCCCGGGGCGCGGCGTCGACGCCATCGCACTGCAGCCGCTGCTG
CATTTCGCGCCGCAGGAGGGCGGCGAGGGCGTGCGGCGCGCGCCGTCCCGGCTG
GCGCAGTGGGACAGCCTCCCCGAGAGCCGCGCGCGCCGGCGCCGCCGCCTGACG
ATGACCATGGGCATGGCGGGCATGGGCGGCATGCCCGGCATGGGGATGGGCGGCA
TGGCCATGAGCATGGGCTTCGACGGCCAGTCCTTCGCCATGGACCGCATCGACCA
GGAGGTGAAGCTGGGCGACGTCGAGATCTGGGAGGTCTCCAGCCAGCCCGGGAT
GATGATGGACATGCAGCATCCCTTCCACATGCACGGCGTGCACTTCGAGGTGCTGC
GGCGCGACGGCGGGCCGCCGGCCGCCCAGGACGCGGGCCGGCGCGACACGGTGC
TGGTGGATGCGCCCGTGCAGCTGCTGGTGCACTTCACGCAGCCGGCCGTCGCGAG
CAGTCCCTTCATGTATCATTGCCACTT

TWGYNGSFLGPTLRLHSGSEARVTVRNRLPEPTTVHWHGLLVPAAVDGGPHNEIAP
DGGMWQPVLPVRQPAATAWYHAHPHMRTAQQVYAGLAGMLIVTDAEEQALGLPSR
YGVDDLPLILQDRFLDGSGRMLYPRGPMTQMHGAFGNTLLVNGAPGPLARVPAALV
RLRLLNAANARSFDLAFARRPQLSQWDRHGKGGLLREPVTRTRLLLAPAQRAELLV
DFSDGAPAMLGTHAAGMLPGRGVDAIALQPLLHFAPQEGGEGVRRAPSRLAQWDS
LPESRARRRRRLTMTMGMAGMGGMPGMGMGGMAMSMGFDGQSFAMDRIDQEVK
LGDVEIWEVSSQPGMMMDMQHPFHMHGVHFEVLRRDGGPPAAQDAGRRDTVLVD
APVQLLVHFTQPAVASSPFMYHCH

>s123-4
ACGTGGGGTTATAACGGTTCGTACCTGGGACCGACGCTGCGTGCCACGCGTGGCG
AGCACGTGCAGGTGAACGTCACCAACGAGGTGGGCGAGACCACGACCACGCACTG
GCACGGGCACCACCTGCCGGCGGAGATGGACGGCGGCCCGCACCAGCCGATCGA
GCCCGGGCAGACCTGGAGTCCGCACTGGACGATCGACCAGCCGGCCGCCACCACC
TGGTACCACCCGCACCTGCACGGCGCCACCGCCGCGCACGTGTACCGCGGCCTGG
CCGGCATGTTCATCCTCGACGACGCCAACAGCCTCGCGCTGGACCTGCCGTCCAC
GTACGGCGTCGACGACATCCCGCTGATCGTGCAGGACCGCGCGTTCGACTCCGCC
GGCCGGGTGGTGGACCGGGCCCCGATGTTCAGCCCGGTCGGGTCGCTGGGCGAC

ACCGTGCTGGCCAACGGCACCGTGGGCGGCTACCTCGAGGTCACCACCGAGCTGG
TGCGGCTGCGGCTGCTCAACGGCTCCAACGCACGGGTCTACGACTTCGGGTTCTC
CGACGGGCGGGAGTTCTCGCTCATCGCCACCGACGGCGGCCTGCTGCCCGCGCCG
CACGTGACCGACCACGTCCAGCTGTCGCCGGGCGAGCGGGCGGAGATCGTGGTG
GCGATGCGGCCGGGCGAGGACGTGGTGCTGCGTAGCCGGGACCCGGACCTCGGC
GCCGGCTTCCTCGAACGGTTCTCCGGCGGGGACGACTCCTTCGACGTCGTGCAGC
TGCGCGCCGCCGACCGGTTGGCACCCTCCCCGCCGCTGCCGCAGCGGCTGGCGCC
GCCGCCGGACCTCGACCCGACGGACGCGCGGGTCACCCGCACGTTCCGGATGGG
GGACATGAACATCAACGGGGCGTCGATGGATATGGGGCGCATCGACGAGGTGGTG
GAGGTCGACAGCACCGAGATCTGGGAGGTCTCCAACGCCGACGGCGTTCCGCACA
GCTTCCACGTCCACGACGTCCAGTTCCGCGTCGTGGCGGTGGACGGCCGGCCGCC
CGGGCCGGAGCTGTCGGGGTGGAAGGACACGGTGTACGTCGCACCGGGTGGCAC
CGTCCGGCTGGTGCTGCGGTTCACCGACTACACCGACCCGGTCTCGCCGTATATGT
ACCACTGCCACAT

TWGYNGSYLGPTLRATRGEHVQVNVTNEVGETTTTHWHGHHLPAEMDGGPHQPI
EPGQTWSPHWTIDQPAATTWYHPHLHGATAAHVYRGLAGMFILDDANSLALDLPST
YGVDDIPLIVQDRAFDSAGRVVDRAPMFSPVGSLGDTVLANGTVGGYLEVTTELVRL
RLLNGSNARVYDFGFSDGREFSLIATDGGLLPAPHVTDHVQLSPGERAEIVVAMRPG
EDVVLRSRDPDLGAGFLERFSGGDDSFDVVQLRAADRLAPSPPLPQRLAPPPDLDPT
DARVTRTFRMGDMNINGASMDMGRIDEVVEVDSTEIWEVSNADGVPHSFHVHDVQ
FRVVAVDGRPPGPELSGWKDTVYVAPGGTVRLVLRFTDYTDPVSPYMYHCH
